# How can human resources for health interventions contribute to sexual, reproductive, maternal, and newborn healthcare quality across the continuum in low- and lower-middle-income countries? A systematic review

**DOI:** 10.1186/s12960-021-00601-3

**Published:** 2021-04-21

**Authors:** Melese Girmaye Negero, David Sibbritt, Angela Dawson

**Affiliations:** 1 School of Public Health, Institute of Health Sciences, Wollega University, Nekemte, Ethiopia; 2 School of Public Health, Faculty of Health, University of Technology Sydney, Sydney, Australia

**Keywords:** Human resources for health intervention, Skilled personnel, Lay personnel, Quality of care, Sexual, Reproductive, Maternal, And newborn health, The continuum of care, Deductive qualitative content analysis, Low- and lower-middle-income countries

## Abstract

**Background:**

Well-trained, competent, and motivated human resources for health (HRH) are crucial to delivering quality service provision across the sexual, reproductive, maternal, and newborn health (SRMNH) care continuum to achieve the 2030 Sustainable Development Goals (SDGs) maternal and neonatal health targets. This review aimed to identify HRH interventions to support lay and/or skilled personnel to improve SRMNH care quality along the continuum in low- and lower-middle-income countries (LLMICs).

**Methods:**

A structured search of CINAHL, Cochrane Library/trials, EMBASE, PubMed, SCOPUS, Web of Science, and HRH Global Resource Centre databases was undertaken, guided by the PRISMA framework. The inclusion criteria sought to identify papers with a focus on 1. HRH management, leadership, partnership, finance, education, and/or policy interventions; 2. HRH interventions' impact on two or more quality SRMNH care packages across the continuum from preconception to pregnancy, intrapartum and postnatal care; 3. Skilled and/or lay personnel; and 4. Reported primary research in English from LLMICs. A deductive qualitative content analysis was employed using the World Health Organization-HRH action framework.

**Results:**

Out of identified 2157 studies, 24 intervention studies were included in the review. Studies where ≥ 4 HRH interventions had been combined to target various healthcare system components, were more effective than those implementing ≤ 3 HRH interventions. In primary care, HRH interventions involving skilled and lay personnel were more productive than those involving either skilled or lay personnel alone. Results-based financing (RBF) and its policy improved the quality of targeted maternity services but had no impact on client satisfaction. Local budgeting, administration, and policy to deliver financial incentives to health workers and improve operational activities were more efficacious than donor-driven initiatives. Community-based recruitment, training, deployment, empowerment, supportive supervision, access to m-Health technology, and modest financial and non-financial incentives for community health workers (CHWs) improved the quality of care continuum. Skills-based, regular, short, focused, onsite, and clinical simulation, and/or mobile phone-assisted in-service training of skilled personnel were more productive than knowledge-based, irregular, and donor-funded training. Facility-based maternal and perinatal death reviews, coupled with training and certification of skilled personnel, positively affected SRMNH care quality across the continuum. Preconception care, an essential component of the SRMNH care continuum, lacks studies and services in LLMICs.

**Conclusions:**

We recommend maternal and perinatal death audits in all health facilities; respectful, woman-centered care as a critical criterion of RBF initiatives; local administration of health worker allowances and incentives; and integration of CHWs into the healthcare system. There is an urgent need to include preconception care in the SRMNH care continuum and studies in LLMICs.

**Supplementary Information:**

The online version contains supplementary material available at 10.1186/s12960-021-00601-3.

## Background

Approximately 810 women die from preventable causes related to pregnancy and childbirth daily, and more than 94% of these deaths occur in LLMICs [1]. In the last two decades, deaths from complications during pregnancy, childbirth, and the postnatal period have declined by 38%. However, an average reduction of 3% per year is too slow to achieve the required SDGs target in 2030 [2]. Avoidable maternal and perinatal morbidity and mortality are attributed mainly to the poor quality of care received in health facilities [3]. In low-income countries, the 2030 SDGs target of reducing the global maternal mortality ratio to less than 70/100,000 live births and the global neonatal mortality rates to less than 12/1000 live births requires a rapid improvement in the quality of SRMNH care. This involves enhancing the availability, skills, and motivation of healthcare providers [4–6].

The SRMNH care continuum is an integrated and continuous care package with evidence-based interventions that are to be delivered over the preconception, pregnancy, birth, and postnatal periods [7]. The recommended preconception care (PCC) services include family planning, abortion care, sexually transmitted diseases prevention and treatment, and health counselling during the pre-pregnancy period [8].

During pregnancy, quality antenatal care (ANC) involves nutritional counselling and multivitamin supplements, adequate visits with skilled personnel (eight and above), blood and urine tests, preventive antibiotics, tetanus toxoid injections, and health education on pregnancy and birth danger signs [9, 10]. Quality intrapartum care (IPC) involves: respectful care, clear and compelling communication between the women and health workers, the option of a companion during labour and childbirth, health facility birth attended by skilled personnel, appropriate pain relief strategies, mobility in labour where possible, and choice of birth position, the use of uterotonics and delayed cord clamping (after a minute), immediate kangaroo care and breastfeeding, delayed bathing of the newborn (24 h), and the care of mother and newborn in a health facility for at least 24 h after birth [10, 11]. Quality postnatal care (PNC) includes immediate PNC within 24 h after birth and at least three additional postnatal visits within 42 days after birth for the mother and newborn, home visits in the first week after birth, exclusive breastfeeding, cord care, prophylactic antibiotics for the mother, and health education on maternal and newborn health danger signs [10, 12].

According to the World Health Organization (WHO), the quality of care provided to women and newborns must be safe, effective, timely, efficient, equitable, and people-centred [3, 13]. Safe care is care that minimizes risk and harm to recipients, including avoiding preventable injuries and reducing medical errors. Effective care focuses on the provision of services that are based on scientific knowledge and evidence-based standards. Timely care avoids harmful delays in giving and receiving care, while efficient care maximizes resource use and avoids wastage. Equitable care does not discriminate based on personal characteristics or socioeconomic status, while people-centred care is care that considers the desire, values, culture, and aspirations of care recipients [3, 13, 14].

According to the WHO, human resources for health are "all people engaged in actions whose primary intent is to enhance health". This includes a range of professionals from clinical to managers, technicians, and researchers [15, 16]. Well-trained, competent, and motivated HRH is crucial to delivering SRMNH care quality across the continuum from PCC to PNC. Therefore, improving health worker performance is key to achieving the SDGs maternal and neonatal health targets [13]. Low-income countries are experiencing a chronic shortage of healthcare providers; many are not geographically distributed according to health service needs and are performing below required standards [6, 17–19]. A systematic review by Lassi et al. conducted in 2016 concluded that improving the management, capacity, and motivation of existing HRH is vital to improving maternal healthcare quality [6].

A synthesis by Munabi-Babigumira et al. (2017) found that pre-service and in-service training, adequate staffing, supervision, incentives, leadership and management support, adequate equipment and supplies, and teamwork and collaboration improved the ability of skilled personnel to deliver quality IPC and PNC services [20]. Althabe et al. (2008) demonstrated that interactive workshops and reminders, educational outreach visits, audit and feedback, mass-media and patient-mediated interventions, financial incentives, and/or organizational and regulatory interventions had a moderately positive effect on healthcare provider performance and the quality of ANC, IPC, PNC, and neonatal care services in low- and middle-income countries [21]. Sibley et al. (2009) found that training in advice on ANC, IPC, and PNC; management of normal delivery; advice on breastfeeding; and timely detection and referral of women with obstetric complications by traditional birth attendants (TBAs) had a positive effect on the increment of timely referrals and reduction of maternal, perinatal and neonatal mortalities, and stillbirth rates [22]. However, there are no reviews examining HRH interventions' contribution to lay and/or skilled personnel's performance to improve SRMNH care quality across the continuum in LLMICs. The review by Lassi et al. focused on skilled personnel only and lacked an examination of HRH interventions contributing to SRMNH care quality across the continuum [6]. We, therefore, undertook an updated and comprehensive review to examine HRH interventions and SRMNH outcomes.

## Methods

**Fig. 1:**
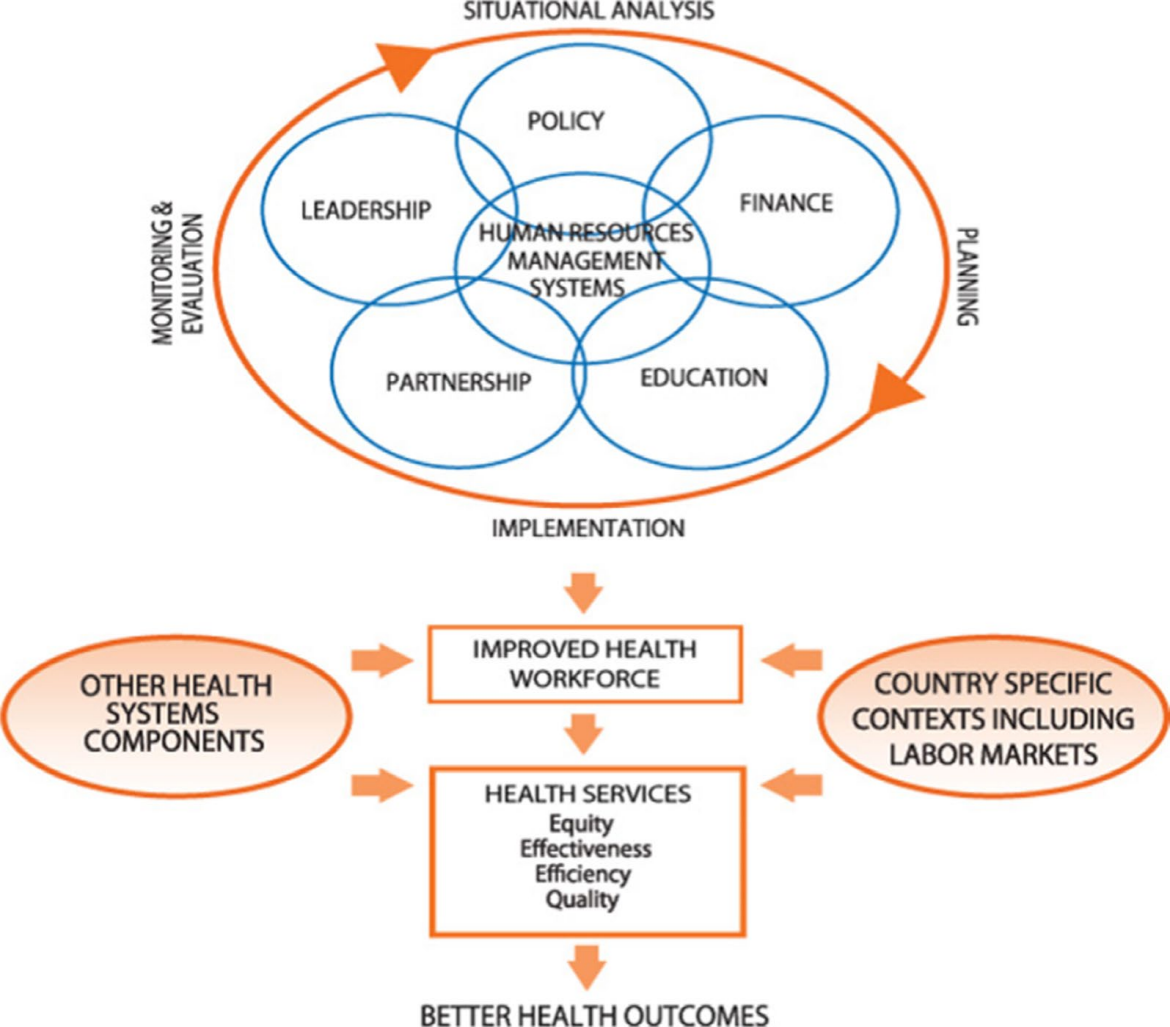
HRH action framework, WHO [25]

A deductive qualitative content analysis of HRH interventions and their effects on SRMNH care quality along the continuum was undertaken using an a priori conceptual framework to help direct and define the study and deliver practical insights for health policy and practice decision-making [23, 24]. We used the WHO-HRH Action Framework [25, 26] to define HRH interventions (Fig. 1). The framework identifies six action fields (Management, Leadership, Partnership, Finance, Education, and Policy) [25, 26]. The Enhancing transparency in reporting the synthesis of qualitative research (ENTREQ) guidance was followed in this review [27], which is registered in the International Prospective Register of Systematic Reviews [28].

### Search protocol

In consultation with two public health research librarians from the University of Technology Sydney (UTS), a Population, Interventions, Comparators, Outcomes, and Study design (PICOS) design, was applied to develop the review question: in LLMICs, how can HRH interventions contribute to SRMNH care quality across the continuum?

Included studies were those that described: (a) an HRH management, leadership, partnership, finance, education, and/or policy intervention (see definitions at Additional file 1: Table S1.); (b) one or more HRH interventions' effect on two or more consecutive quality SRMNH care packages across the continuum (a study reporting HRH interventions delivered in conjunction with one or more non-continuous SRMNH care packages was excluded since it was not a continuum); (c) the role of skilled and/or lay personnel (see definitions at Additional file 1: Table S2.); and (d) primary research studies in English (other languages were excluded due to resource constraints) conducted in LLMICs (see inclusion criteria at Additional file 1: Table S3.). Studies that did not include any of the six HRH interventions were excluded. We defined a quality SRMNH care package as one that contained a safe, effective, timely, efficient, equitable, and people-centred package of interventions that comprised PCC, ANC, IPC, and/or PNC (maternal and/or newborn) services as described by the WHO [8, 13]. As noted in the protocol for this review, the main outcome of the review is quality of care (defined according to WHO as care that is safe, effective, timely, efficient, equitable, and people-centered). Additional outcomes included were maternal and/or neonatal health outcomes directly related to the quality of care. These involve maternal and/or neonatal morbidity or mortality and uptake of the recommended and life-saving interventions facilitated by health workers (such as early initiation of breastfeeding, delayed bathing of newborns, and cord care) [28]. 

### Search strategy

**Fig. 2:**
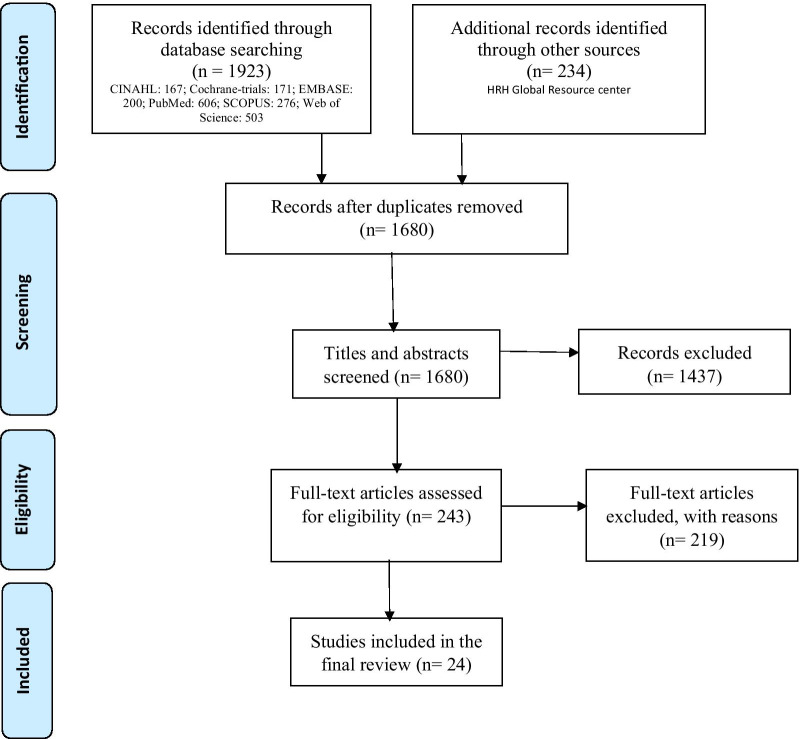
Overview of the literature review process, PRISMA 2009 [29]

Six bibliographic databases (PubMed, Web of Science/Core Collection, SCOPUS, CINAHL, EMBASE/OVID, and Cochrane Library/trials) were systematically searched in consultation with two public health research librarians from the UTS (Additional file 1: Table S4.). The search engine: HRH Global Resource Centre was searched for grey literature. Quantitative, qualitative, and mixed-method studies published between 01 January 2000 and 31 December 2019 were retrieved. This period was chosen to evaluate progress over time in SRMNH care quality along the continuum in relation to HRH interventions since the end of the Alma Ata Health for All Declaration in 2000, the Millennium Development Goals (MDGs) era, and in the first 5 years of implementation of the SDGs. The Preferred Reporting Items for Systematic Reviews and Meta-Analyses (PRISMA) 2009 guideline was used to outline the review process [29]. A total of 2157 studies were identified, including 1923 from six bibliographic databases and 234 from the HRH Global Resource Centre (Fig. 2), with 477 duplicates being identified and removed using the endnote de-duplication guidelines [30].

Two reviewers (MG and AD) independently used the Covidence online production tool for the title and abstract screening, full-text screening, data abstraction, and quality assessment. Differences in decisions regarding the final papers for review and quality assessment were resolved through a review by the third author (DS), and a consensus was reached. A total of 1437 and 219 articles were excluded during the title and abstract screening and full-text eligibility assessment, respectively, because they did not meet the inclusion criteria.

### Quality appraisal

Two reviewers (MG and AD) independently appraised the methodological quality of 25 studies that met the inclusion criteria to describe their methodological quality and ensure that there was enough methodological detail to ensure rigour to be included for the review. The Cochrane methods were applied to appraise the quality of randomized controlled trials (RCTs) (Additional file 1: Table S5) [31]. Each RCT was evaluated for internal validity and quality of reporting. Quality of quasi-experimental, prospective (pre/post), post-only and comparison, and post-only studies were appraised using the Joanna Briggs Institute (JBI) critical appraisal checklist for quasi-experimental studies (non-randomized experimental studies) (Additional file 1: Table S6) [32]. Studies collecting qualitative or mixed data were appraised using the United Kingdom's National Health Service Critical Appraisal Skills Programme (CASP) qualitative checklist (Additional file 1: Table S7) [33]. We included intervention studies with a methodologically low risk of bias or moderate to high quality for the review. One pre/post-test study was excluded during the quality appraisal because it has a low-quality score (3 out of 9). Using the quasi-experimental studies' quality appraisal tool, a study with "yes" responses for the 9 signalling questions of less than 4 was considered low quality [34].

### Data abstraction and synthesis

Data from the 24 studies were systematically extracted into tables using templates based on the Cochrane methods to integrate qualitative and implementation evidence within intervention effectiveness reviews [35].

A deductive qualitative content analysis of the extracted text related to each implemented HRH intervention from each included study was undertaken through coding texts according to emergent descriptions and labelling and structured along with the four categories of SRMNH care continuum [36, 37]. Tables and concept maps were used to plot patterns and relationships across these categories, and robustness was assessed through critical reflection and discussion between the three authors.

## Results

Twenty-four intervention studies were included in this review and are summarized in Table 1. According to the World Bank Country and Lending Groups' Classification 2019–2020 [38], 11 studies were from low-income countries [39–49], 11 were from lower-middle-income countries [50–60], and two were conducted in both low-income and lower-middle-income countries [61, 62] (Table 2).

**Table 1:** Summary of studies about effects of HRH interventions on SRMNH care quality across the continuum in LLMICs, 2020

References	HRH interventions						The SRMNH care continuum			
	Policy	Finance	Education	Partnership	Leadership	Management	PCC	ANC	IPC	PNC
Agarwal et al. (2019) [54]	X		X			X		X	X	X
Ayalew et al. (2017) [41]		X	X	X	X	X		X	X	X
Balakrishnan et al. (2016) [55]		X	X	X		X		X	X	X
Basinga et al. (2011) [44]	X	X		X	X	X		X	X	
Binyaruka et al. (2015) [46]	X	X			X			X	X	X
Bonfrer et al. (2014) [40]	X	X				X		X	X	
Duysburgh et al. (2016) [61]	X	X		X		X		X	X	
Edwards et al. (2011) [50]			X	X	X	X		X	X	X
Engineer et al. (2016) [39]	X	X					X	X	X	X
Ghosh et al. (2019) [56]			X	X		X			X	X
Gomez et al. (2018) [52]	X	X	X			X			X	X
Kambala et al. (2017) [42]	X	X	X	X				X	X	X
Larson et al. (2019) [47]			X			X		X	X	
Magge et al. (2017) [45]		X	X	X	X			X	X	X
Maru et al. (2017) [43]	X	X		X		X		X	X	X
McDougal et al. (2017) [57]	X	X	X	X		X		X	X	X
Mwaniki et al. (2014) [58]	X			X	X	X		X	X	
Okawa et al. (2019) [53]		X	X		X	X		X	X	X
Okuga et al. (2015) [48]	X	X	X	X	X	X		X	X	X
Pirkle et al. (2013) [62]		X	X	X	X	X			X	X
Rahman et al. (2011) [51]		X	X	X				X	X	X
Satti et al. (2012) [59]	X	X	X	X		X		X	X	
Waiswa et al. (2015) [49]	X	X	X	X		X		X	X	X
Zeng et al. (2018) [60]	X	X					X	X	X	X
Total number of Xs	15	19	16	16	9	18	2	21	24	18

**Table 2:** Human resources for health interventions and their effects on SRMNH care quality across the continuum in low- and lower-middle-income countries, 2020

References	Contex*ts*	HRH interventions	Duration of the interventions	Type of health workers	Intervention settings	Methods	Effects of the interventions	Quality of the study
Agarwal et al. (2019) [54]	India	Training and deployment of lay personnel to provide: health education, linkage of women to healthcare facilities, and home-based ANC and PNC services	06 years	Accredited Social Health Activists (ASHAs)	Primary care (Community based)	Indian Human Development Survey (IHDS)-II (2011–2012 data): secondary data analysis	Exposure to ASHA agents: significantly associated with ANC 1 and SPAB use across the continuum; no significant impact on ≥ 4 ANC or PNC use between exposed and non-exposed women; 12% increase in women receiving at least some of the services; 8.8% decrease in women receiving no services; it is not significantly associated with completion of all services along the continuum	Moderate
Ayalew et al. (2017) [41]	Ethiopia	Standards-Based Management and Recognition (SBM-R) approach (multi-faceted interventions): BEmONC training; supportive supervision; audit and site mentoring; sector-wide leadership; quality improvement team in each facility; mobilizing financial resources; and community involvement	03 years	Doctors, health officers, midwives, and nurses	Primary healthcare (8 Health centres) and 3 secondary care Hospitals	A post-only intervention versus comparison facilities design: observations of service delivery using structured checklists to measure provider performance in ANC, uncomplicated labour and delivery care, and immediate PNC	A significant difference of 22 pp for each newborn and mother PNC skill area; significant positive impact on maternal and newborn health providers' performance during labour and delivery and immediate PNC services, but not during ANC services	High
Balakrishnan et al. (2016) [55]	India	Mobile technology—a health system strengthening multi-stakeholder cooperation (mHealth platform): community-based frontline health workers training on mHealth platform and provision of maternal and child healthcare; supportive supervision; and mobilizing financial resources	02 years	ASHAs, Anganwadi workers, auxiliary nurse-midwives, and lady health supervisors	Primary care (community based)	A quasi-experimental study with pre- and post-implementation evaluation at intervention, and control areas: coverage of quality indicators of maternal–child healthcare continuum compared with control area and the previous year	Implementation blocks had higher coverage of all the 07 quality indicators as compared to the control and the previous year—intervention area vs previous year vs control: registration within the 1st trimester (15% vs 10% vs 10%), complete ≥ 3 ANC visits (56% vs 51% vs 48%), at least 1TT vaccine (79% vs 74% vs 80%), ≥ 90 Iron and Folic Acid Tablets (62% vs 50% vs 49%), health facility birth (84% vs 59% vs 67%), breastfeeding within 1 h of birth (98% vs 73% vs 73%), at least 1 PNC home visit (28% vs 18% vs 10%); there was equity of services across castes for all indicators—scheduled castes/tribes vs other castes: registration within the 1st trimester (15% vs 15%), complete ≥ 3 ANC contacts (55% vs 56%), at least 1 TT vaccine (77% vs 79%), ≥ 90 Iron and Folic Acid Tablets (60% vs 62%), health facility birth (78% vs 87%), breastfeeding within 1 h of birth (95% vs 95%), at least 1 PNC home visit (29% vs 28%); timely capture of data compared to paper-based reporting: average time lag of 72 days (≈ 2.5 months) is overcome by instant data capture with the mHealth platform	High
Basinga et al. (2011) [44]	Rwanda	Quarterly performance-based payment for healthcare providers, directly observed supervision, leadership, and hospital team advisory group	18–23 months	Doctors and mid-level cadres	Primary care (Primary health centres)	Prospective impact evaluation between P4P facilities (intervention) and traditional input-based funding facilities (controls); baseline and end-line surveys at facilities and households; difference-in-differences analysis (DiD) where p-value was the cluster-adjusted t-test	Greatest effect on indicators that had the highest payment rates and needed the least effort from the service provider: an increase of 0·157 standard deviations (*p* = 0·02) in ANC quality (against Rwandan prenatal clinical care practice guidelines), 7.2% increase in Tetanus vaccine injections during ANC (*p* = 0·057), no improvements in ≥ 4 ANC coverage (*p* = 0·825), 23% increase in facility birth in the intervention group (*p* = 0·017)	High
Binyaruka et al. (2015) [46]	Tanzania	Biannual P4P for health workers and district and regional health managers targeting eight specific MCH care services, leadership	13 months	Skilled personnel	Primary healthcare (health centres, faith-based and parastatal dispensaries, and public dispensaries) and secondary care hospitals	A Controlled Before and After household and facility survey study: DiD analysis (effect-*β*)	A 0.05 (*β*: 0.05; *p* = 0.03) increase in the patient satisfaction score for non-targeted services, a 5.0% reduction in out-of-pocket payment for birth (*β*: − 5.0; *p* = 0.023). No evidence of effect of P4P on patient experience of care for targeted services: at least 2 doses of intermittent preventive malaria treatment (IPT) during ANC (*p* = 0.001), HIV treatment during ANC (*p* = 0.893), health facility birth (*p* = 0.001), polio vaccine at birth (*p* = 0.093), PNC (*p* = 0.823), postnatal family planning (*p* = 0.844), ANC contents (*p* = 0.118), interpersonal care satisfaction during birth (*p* = 0.505), staff kindness during birth (*p* = 0.088), waiting time (*p* = 0.636), consultation time (*p* = 0.650)	High
Bonfrer, Poel and Doorslaer (2014) [40]	Burundi	Performance-based financing (PBF); quarterly quality assessment by local regulatory authorities	01–04 years	Doctor, nurse, and midwife	Primary healthcare facilities	Burundi Demographic and Health Survey-BDHS (2010–2011) data; the difference-in-differences analysis; provinces with PBF vs. without PBF	No significant effect on first-trimester ANC, ≥ 1 ANC visit or BP measurement during pregnancy; significant impact with 10 pp increase (*p* < 0.001) on ≥ 1 anti-tetanus vaccination during ANC and with 5 pp increase on SPAB for women where PBF was in place from the start of pregnancy; no significant effect on neonatal mortality; no impact on equitable care: higher probability of BP measurement during pregnancy among non-poor; a significant increase in SPAB (*p* < 0.028) among the non-poor, and no effect on SPAB among the poor	High
Duysburgh et al. (2016) [61]	Rural Burkina Faso, Ghana, and Tanzania	A computer-assisted clinical decision support system (eCDSS) and performance-based incentives: performance productivity; job satisfaction; financial and non-financial incentives; incentive policies; local research stakeholder cooperation (eCDSS maintenance)	02 years	Medical officer, assistant medical officer, clinical officer, assistant clinical officer, nurse/midwife and auxiliary nurse/midwife	Rural primary healthcare facilities	An intervention study: 06 intervention and 06 non-intervention PHC facilities in each country; assessment of quality of care in each facility by health facility surveys, direct observation of antenatal and childbirth care, patient satisfaction exit interviews, and reviews of patient records and maternal and child health registers; pre- vs. post-intervention and intervention vs. non-intervention health facilities' quality assessment	No significant difference in quality scores of ANC and delivery care to pre-intervention time or non-intervention facilities’ scores. Total ANC observation quality scores (pre- vs post-intervention: 0.83 vs 0.87, *p* = 0.06; intervention vs non-intervention facilities at end line: 0.87 vs 0.86, *p* = 0.33); total ANC satisfaction survey quality scores (pre- vs post-intervention: 0.42 vs 0.71, *p* = 0.09; intervention vs non-intervention facilities at end line: 0.71 vs 0.55, *p* = 0.73); total ANC patient record review quality scores (pre- vs post-intervention: 0.75 vs 0.82, p = 0.03; intervention vs non-intervention facilities at end line: 0.82 vs 0.71, *p* = 0.03); total childbirth observation quality scores (pre- vs post-intervention: 0.65 vs 0.75, *p* = 0.01; intervention vs non-intervention facilities at end line: 0.75 vs 0.68, *p* = 0.11); total childbirth satisfaction survey quality scores: (pre- vs post-intervention: 0.70 vs 0.84, p = 0.71; intervention vs non-intervention facilities at end line: 0.84 vs 0.83, *p* = 0.90); total BEmONC signal functions scores (pre- vs post-intervention: 0.75 vs 0.72, *p* = 0.09; intervention vs non-intervention facilities at end line: 0.72 vs 0.68, *p* = 0.97); history taking on vaginal bleeding—ANC observation scores (pre- vs post-intervention: 0.22 vs 0.32, *p* = 0.17; intervention vs non-intervention facilities at end line: 0.32 vs 0.19, *p* = 0.02); counselling on vaginal bleeding—ANC observation scores (pre- vs post-intervention: 0.49 vs 0.43, p = 0.65; intervention vs non-intervention facilities at end line: 0.43 vs 0.42, p = 0.80); history taking on vaginal bleeding—childbirth observation scores (pre- vs post-intervention: 0.32 vs 0.32, *p* = 0.20; intervention vs non-intervention facilities at end line: 0.32 vs 0.31, *p* = 0.54); administer oxytocin after childbirth—childbirth observation scores (pre- vs post-intervention: 0.92 vs 0.96, *p* = 0.70; intervention vs non-intervention facilities at end line: 0.96 vs 0.91, p = 0.72); monitoring uterine retraction after childbirth—childbirth observation scores (pre- vs post-intervention: 0.41 vs 0.60, *p* = 0.02; intervention vs non-intervention facilities at end line: 0.60 vs 0.56, *p* = 0.17); assessing vaginal bleeding after childbirth—childbirth observation scores (pre- vs post-intervention: 0.60 vs 0.78, *p* = 0.10; intervention vs non-intervention facilities at end line: 0.78 vs 0.72, *p* = 0.38); ANC satisfaction scores—counselling on vaginal bleeding (pre- vs post-intervention: 0.44 vs 0.47, *p* = 0.91; intervention vs non-intervention facilities at end line: 0.47 vs 0.17, *p* = 0.79); women who received oxytocin (%)—childbirth record review (pre- vs post-intervention: 89 vs 89, *p* = 0.96; intervention vs non-intervention facilities at end line: 89 vs 96, *p* < 0.01); checking BP—ANC observation scores (pre- vs post-intervention: 0.98 vs 0.97, *p* = 0.96; intervention vs non-intervention facilities at end line: 0.97 vs 0.93, *p* = 0.77); lab proteinuria examination—ANC observation scores (pre- vs post-intervention: 0.32 vs 0.47, *p* = 0.03; intervention vs non-intervention facilities at end line: 0.47 vs 0.22, *p* < 0.01); counselling on hypertensive disorders danger signs—ANC observation scores (pre- vs post-intervention: 0.45 vs 0.43, *p* = 0.68; intervention vs non-intervention facilities at end line: 0.43 vs 0.37, *p* = 0.57); monitoring BP—childbirth observation scores (pre- vs post-intervention: 0.53 vs 0.68, p = 0.04; intervention vs non-intervention facilities at end line: 0.68 vs 0.62, *p* = 0.30); counselling on hypertensive disorder danger signs—ANC satisfaction scores (pre- vs post-intervention: 0.02 vs 0.42, *p* = 0.17; intervention vs non-intervention facilities at end line: 0.42 vs 0.19, *p* = 0.90); lab proteinuria exam—ANC record review (pre- vs post-intervention: 0.51 vs 0.69, *p* = 0.11; intervention vs non-intervention facilities at end line: 0.69 vs 0.29, p = 0.01); partograph correctly used—childbirth observation (pre- vs post-intervention: 0.58 vs 0.75, *p* = 0.03; intervention vs non-intervention facilities at end line: 0.75 vs 0.60, *p* = 0.15); deliveries with correctly completed partograph (%)—record review (pre- vs post-intervention: 42 vs 70, *p* < 0.01; intervention vs non-intervention facilities at end line: 70 vs 48, *p* < 0.01)	High
Edwards and Sahab (2011) [50]	Rural Bangladesh	Skills-based training; collaboration and teamwork at all levels; community involvement; monthly supportive supervision; leadership	06 years	Village health volunteers, community health workers, community health assistants, and community skilled personnel	Primary care (healthcare centres and community based), and General hospital (Comprehensive essential obstetric and newborn care)	Country case study: Lutheran Aid to Medicine in Bangladesh (LAMB) Integrated Rural Maternal and Child Healthcare' Home-to-Hospital, Continuum-of-Care' approach	LAMB areas vs. national sample: care received by women (≥ 1 ANC: 81% vs. 52%; SPAB: 32.2% vs. 18%; caesarean section rate: 4.8% vs. 2.7%; and PNC: 85% vs. 22%); a higher proportion of poor women (in wealth quintile-1) received ANC, SPAB, caesarean section, and PNC; the gap in service use between the poorest and the richest women is much smaller	Moderate
Engineer et al. (2016) [39]	Afghanistan	Quarterly Pay-for-Performance (P4P) for health workers; mobilizing financial resources	23–25 months	Skilled personnel	Primary healthcare facilities	A cluster-randomized trial: end line household survey and quality assessment in health facilities in P4P and comparison areas	The P4P had no significant impact on increasing coverage or equity (by wealth index) of targeted MCH services at population level (P4P vs comparison): modern contraception (10.7% vs 11.2%; *p* = 0.90); ANC (56.2% vs 55.6%; *p* = 0.94); SPAB (33.9% vs 28.5%, *p* = 0.17); PNC (31.2% vs 30.3%, *p* = 0.98); equity in SPAB /concentration index (0.1758 vs 0.1000; *p* = 0.3); Quality of care (P4P vs comparison): Overall Client Satisfaction and Perceived Quality of Care Index (76.5% vs 75.1%; *p* = 0.2); Health Worker Satisfaction Index (63.8% vs 63.4%; *p* = 0.9); Health Worker Motivation Index (72.7% vs 72%; *p* = 0.4); quality of care/ history taking and physical examinations index (76.4% vs 72.3%; p = 0.01); quality of care/client counselling index (35.3% vs 29.3%; p = 0.01); quality of care/time spent with client index (14.5% vs 8.6%; p = 0.05)	Selection: LR Performance: LR Attrition: LR Detection: LR Reporting: LR
Ghosh R. et al. (2019) [56]	India	Multi-faceted onsite nurse mentoring and simulation (diagnosis and management of intrapartum asphyxia and PPH): skills demonstrations, didactic sessions, high-fidelity simulation, bedside mentoring, and team training during actual patient care were the mentoring activities; weekly nurse-mentoring, PRONTO International's simulation, team training; NGO collaboration	20 months	Auxiliary Nurses and general nurse-midwives	BEmONC facilities at Primary care	A quasi-experimental (b/n facilities) and a longitudinal (within facilities) comparison studies over time	Between-facility comparisons across phases: diagnosis was higher in final week of intervention (intrapartum asphyxia: 4.2–5.6%, PPH: 2.5–5.4%) relative to the 1^st^ week (intrapartum asphyxia: 0.7–3.3%, PPH: 1.2– 2.1%); within-facility comparisons: intrapartum asphyxia Dx among all live births increased from 2.5% in week-1 to 4.8% in week-5, after which it reduced to 4% through week-7, PPH Dx increased from week-1 through 5 (from 1.6% to 4.4%) after which it decreased through week-7 (3.1%); facility performance index—on a scale of 100 from baseline (1st 3 wks.) to end line (≥ 4 wks.): median intrapartum care score (IQR) = [21 (8–29)—58 (42–67)], median newborn care score (IQR) = [42 (35–50) 71 (58–79)]; diagnosis per additional week of mentoring, adjusted incidence rate ratios (IRR, 95% CI): asphyxia (Wks. 1–5: 1.21(1.13, 1.29), *p* < 0.001; wks. 5–7: 0.91(0.82, 1.01), *p* = 0.073; PPH (Wks. 1–5: 1.17 (1.05, 1.31), *p* = 0.006; wks. 5–7: 0.86 (0.77, 0.97), *p* = 0.017; management per additional week of mentoring (IRR, 95%CI): asphyxia [(radiant warmer: 1.05 (1.01, 1.09), *p* = 0.005; drying-stimulation: 1.05 (1.02, 1.08), *p* = 0.003; suctioning: 1.03 (0.99, 1.06), *p* = 0.127; positive pressure ventilation (PPV):1.09 (1.02, 1.15), *p* = 0.007] and PPH [IV fluids: 1.01 (0.97, 1.04), *p* = 0.688; uterotonics: 0.99 (0.95, 1.03), *p* = 0.700]	High
Gomez et al. (2018) [52]	Ghana	On-site, low-dose, high-frequency training in BEmONC of registered or certified skilled personnel: two 4-day low-dose sessions, high-frequency practice sessions using anatomic models and mentoring with SMS reminder messages and quizzes; clinical simulation; follow-up mentorship and appraisal (mobile or onsite); mobilizing financial resources	18 months	Midwives	40 secondary care public and missionary hospitals	A cluster-randomized trial: prospective intrapartum stillbirths and 24-h newborn mortality for 12 months. Baseline mortality rates were collected retrospectively 6 months pre-intervention	36% reduction (ARR: 0.64; 95% CI: 0.53–0.77; *p* < 0.001) in 1st 1–6 months of implementation and 52% reduction (ARR: 0.48; 95% CI: 0.36–0.63; *p* < 0.001) in second 7–12 months of implementation in intrapartum stillbirth rates as compared to pre-intervention period, respectively; 59% reduction (ARR: 0.41; 95% CI: 0.32–0.51; *p* < 0.001) in 1st 1–6 months and 70% reduction (ARR: 0.30; *p* < 0.001) in 2nd 7–12 months in 24-h newborn mortality rates as compared to pre-intervention period, respectively	Selection: SC Performance: LR Attrition: LR Detection: LR Reporting: LR
Kambala et al. (2017) [42]	Rural Malawi	RBF for Maternal and Newborn Health initiative: quarterly performance-based financing (supply-side financial incentive upon attainment of a pre-defined set of indicators, 70% for staff bonuses and 30% for health facility’s operational activities, health management teams were rewarded with financial incentives based on the overall performance of a district as a measure of the adequacy of supervision) and financial incentives to women for delivering in a health facility (demand-side incentive, conditional cash transfers to mothers for giving birth in a health facility); health workers advisory group; mobilizing financial resources; refresher in-service training on antenatal management, obstetric care, and quality assurance; RBF policy	03 years	Healthcare managers, skilled personnel	33 primary and secondary EmOC facilities (Basic and comprehensive)	Mixed method prospective sequential controlled pre- and post-test study over intervention vs. control facilities: client exit interviews, in-depth interviews and FGDs with women and In-depth interviews with health service providers; difference-in-differences analysis (DiD)	End-term vs baseline cohorts (DiD adjusted): mean effect estimate of women’s perceptions on interpersonal relations (ANC: − 0.2, *p* = 0.56; L&D: − 0.1, *p* = 0.70; PNC: − 0.3, *p* = 0.45); mean effect estimate of women’s perceptions on quality of amenities (ANC: − 0.2, *p* = 0.54; L&D: − 0.3, p = 0.45; PNC: − 0.49, *p* = 0.14); mean effect estimate of women’s perceptions on technical care (ANC: − 0.2; *p* = 0.39; L&D: − 0.1, p = 0.85; PNC: − 0.31, *p* = 0.38). No significant effect on women’s perceptions of technical care, quality of amenities, and interpersonal relations for any of the three sets of services observed (ANC, L&D, and PNC); increased the proportion of women reporting to have received medications/treatment during childbirth. Qualitative interviews: most women reported improved health service provision as a result of the intervention; drugs, equipment, and supplies were readily available due to the RBF4MNH; instances of neglect, disrespect, and verbal abuse during the process of care; increased workload resulting from an increased number of women seeking services at RBF4MNH facilities	Moderate
Larson et al. (2019) [47]	Rural Tanzania	In-service training; mentoring; supportive supervision; peer outreach	04 years	Mid-level cadres	Primary care (community-based and primary care clinics)	A cluster-randomized study: baseline (2012) and end line (2016) household surveys in control and intervention catchments; difference-in-differences analysis (DiD)	Total study population-DiD: improved quality of ANC/contents of ANC [Adjusted (A) RR: 1.64; 95% CI: 1.00–2.71]; perceived quality of ANC (ARR: 1.14; 95% CI: 0.88–1.47); perceived obstetric care quality at intervention facility (ARR: 1.13; 95% CI: 0.79–1.62); reduced payment for obstetric care at intervention facility (ARR: − 3.76; 95% CI: − 7.02 to − 0.49). Previous home births-DiD: improved quality of ANC/contents of ANC (ARR: 2.31; 95% CI: 1.44–3.71); improved perceived quality of ANC (ARR: 1.57; 95% CI: 1.07–2.31); perceived obstetric care quality at intervention facility (ARR: 1.12; 95% CI: 0.78–1.59); reduced payment for obstetric care at intervention facility (ARR: − 2.24; 95% CI -4.76—0.28)	Selection: LR Performance: SC Attrition: LR Detection: LR Reporting: LR
(Magge et al. (2017) [45]	Rwanda	Monthly onsite, regular clinical mentorship and training on evidence-based life-saving maternal and newborn care; learning collaborative to build healthcare workers’ leadership in data utilization for continuous quality improvement (QI); mobilizing financial resources; procurement and distribution of essential equipment and supplies	18 months	Nurses, community health supervisors, data officers, and health facility and district leadership	Primary care (Community-based and health centres), and secondary care hospitals	A retrospective case study using the quantitative method: pre–post intervention evaluation	Pre- vs post-intervention: ≥ 4 ANC (23% vs 38%); 1st trimester ANC (23% vs 34%); pregnant women with premature rupture of membrane (PROM) treated with antibiotics (24% vs. 38%); pregnant women with preterm labour treated with corticosteroids (26% vs 75%); SPAB (87% vs. 95%); time to C-section in minutes [median, (IQR): 99 (50–195) vs. 72 (59–77)]; immediate skin-to-skin care after delivery (19% vs. 87%); newborns checked for danger signs within 24 h of birth (47% vs. 98%)	Moderate
Maru et al. 2017) [43]	Rural, remote Nepal	Accountable public–private partnership through integrating community health workers into facility-based care: CHWs conduct surveillance of conditions in the community, triage, referral, and care coordination with healthcare facilities; government’s performance-based accountable payment	18 months	Community health workers	Primary healthcare (Community-based, village clinics/health posts) and secondary care district hospitals	A prospective pre–post pilot study: a household-level census survey to compare population-level maternal, newborn, and child healthcare indicators to the baseline	Pre- vs post-intervention: ≥ 4 ANC [(Increased by 6.4 pp); coverage increased (83% vs 90%)]; health facility birth [(increased by 11.8 pp; *p* < 0.001); coverage increased (81% vs 93%)]; postnatal contraception [(rate increased by 27.5 pp; *p* < 0.001); coverage increased (19% vs 47%)]; infant mortality rate (18.3/1000 vs 12.5/1000); 95% received ultrasound examination by month 8 or 9 of pregnancy	Moderate
McDougal et al. (2017) [57]	India	Training, mobilizing, monitoring, and empowering government Frontline workers (FLWs) and community outreach (home-based) interventions: job aids and tools; mobile service training course for FLWs to expand and refresh their knowledge of life-saving RMNCH behaviours; community involvement; mobilizing financial resources; local policy	02 years	ASHAs, auxiliary nurse midwives, and Anganwadi (Social Service) workers	Primary care (community-based and primary healthcare facilities)	A two-armed quasi-experimental study (intervention vs. control areas); house to house survey of women aged 15–49 with a 0–5-month-old child at baseline and follow-up; difference-in-differences (DiD) analyses	The mean number of services/behaviours used along the RMNH continuum of care (CoC) was significantly higher in intervention areas as compared to control areas at follow-up (0.94 vs. 0.51 health services/behaviours; *p* < 0.0001); overall RMNH CoC coverage in intervention areas increased by 0.41 (Coefficient: 0.41; 95% CI 0.24–0.59; *p* < 0.001) health services/behaviours as compared to the control areas: DiD: ≥ 4 ANC (*p* = 0.23); SPAB (*p* = 0.98); nothing applied to the cord (*p* = 0.01); skin-to-skin care (*p* = 0.03); first bath delayed by ≥ 2 days (*p* = 0.26); breastfed child within 1 h of birth (*p* = 0.39); PNC visit for mother or baby within 48 h (*p* = 0.69); postpartum contraception (*p* < 0.01); child exclusively breastfed (*p* = 0.47); gender equity interaction analysis showed diminished intervention effects on ANC, SPAB and exclusive breastfeeding for women married as minors	High
Mwaniki et al. (2014) [58]	Rural Kenya	Quality improvement ‘collaborative’ health worker advising: regular meeting of a group of health workers from different health facilities that work on the same set of quality indicators to examine performance gaps in service delivery, the causes of these gaps, and solutions to address them; employee relations; leadership; community involvement	20 months	Healthcare managers, skilled personnel, community health workers, and traditional birth attendants	Primary care (3 health centres and 17 dispensaries), and 1 government-run secondary care hospital	A pre- and post-implementation evaluation: data were collected and entered into routine govt. registers daily by the teams and were then used to evaluate 20 indicators of care quality improvement activities monthly	ANC visits in the first trimester (< 16 weeks G.A) increased significantly (8% to 24%; *p* = 0.002), and those making ≥ 4 ANC visits significantly increased (37% to 64%; *p* < 0.001); ANC visits per month with standardized care substantially increased (< 40% to 80–100%; *p* < 0.001) within 03 to 06 months; SPAB significantly increased per month from (33% to 52%; *p* = 0.012); pregnant women actively referred from the community (by community representatives) to health facilities for ANC, and birth care significantly increased (13 per month to 81 per month; *p* < 0.001)	Moderate
Okawa et al. (2019) [53]	Rural Ghana	Orientation of supervisors and healthcare providers in the continuum of care (CoC); distribution of CoC cards to women, home visits to provide PNC within 48 h for those who missed the first 24 h visit; mobilizing financial resources; monthly supervision and monitoring; capacity building to lead sector-wide collaboration	12 months	Doctor, midwife, nurse, community health officer, and community health nurse, and health assistant	Primary healthcare (community-based, private clinics, health centres) and secondary care district hospital	A cluster randomized controlled trial: baseline and follow-up survey to measure adequate contacts (≥ 4 ANC, SPAB, and three timely contacts within 6 weeks postnatal) and quality care (six components during ANC, 3 during peripartum care (PPC), and 14 during postnatal); difference-in-differences analysis (DiD)	The interventions improved contacts with healthcare providers and quality of care during PNC, not in ANC or IPC, regular contacts with healthcare providers did not guarantee quality of care: 12.6% of women in the intervention group received all 6 items during ANC (4.9% baseline), 33.6% received all 3 items during PPC (23.8% baseline) and 41.5% of women and their newborns received all 14 items during PNC (11.5% baseline); adjusted DiD estimators: no significant changes across the three phases: ANC (*p* = 0.61), PPC (*p* = 0.69) and PNC (*p* = 0.35); the percentage of adequate contacts with high-quality care in the intervention group in the follow-up survey and the adjusted DiD estimators (with baseline adequate contacts for ANC, PPC and PNC of 4.9%, 20.2% and 1.3%, respectively) were 12.6% and 2.2 (*p* = 0.61) at ANC, 31.5% and 1.9 (*p* = 0.73) at PPC and 33.7% and 12.3 (*p* = 0.13) at PNC in the intention-to-treat design (real world-effectiveness of the intervention), whereas 13.0% and 2.8 (*p* = 0.54) at ANC, 34.2% and 2.7 (*p* = 0.66) at PPC and 38.1% and 18.1 (*p* = 0.02) at PNC in the per-protocol design (ideal world-designated by possession of continuum-of-care card); in intention-to-treat design, 76.9% of women in the intervention group in the follow-up survey had adequate contacts during ANC; however, only 12.6% had quality-adjusted adequate contacts; 82.0% SPAB, while only 31.5% had SPAB with high-quality care; during PNC, 62.2% of women and their newborns had adequate contacts, however, only 33.7% had quality-adjusted adequate PNC contacts	Selection: LR Performance: LR Attrition: LR Detection: LR Reporting: LR
Okuga et al. (2015) [48]	Uganda	Recruitment, training, immediate deployment and incentivization of CHWs; skilled personnel’s in-service training and provision of essential equipment and supplies: selected by the community; 07 days training on identifying pregnant women, and make two pregnancy home visits and three postnatal home visits in the first week after birth; financial and non-financial incentives (t-shirt, briefcase and certificate, and transport allowance); directly observed supervision visits by nurses/midwives and group supervision meetings monthly then quarterly; mobilizing financial resources	02 years	Community health workers and skilled personnel	Primary care (community-based and primary healthcare facilities)	A community-based cluster-randomized control trial: in-depth interviews (IDIs) and focus group discussions (FGDs) involving facility-based health workers, members of the District Health Team, village leaders, mothers with children less than 6 months of age, and CHWs both from urban and rural areas	CHWs highly appreciated in the community and seen as important contributors to maternal and newborn health at a grassroots level; more women attending ANC during the first trimester; husbands/partners save money, provide women with money for emergencies, transport, and babies’ needs; women attend to their health needs during pregnancy; women recognize danger signs; more births at health facilities; women experience a caring attitude from health workers; women with CHW referral slips are seen faster at hospital or health unit; women put only salty water on the baby’s umbilical cord rather than animal dung and herbs; bathing is delayed instead of immediately practiced; more women taking their newborn babies to health facilities for PNC and immunization; immediate breastfeeding at birth and continuous breastfeeding; more women giving colostrum	Moderate
Pirkle et al. (2013) [62]	Mali and Senegal	Maternal death review (auditing maternal deaths in the facility), workshops on obstetrical best experiences, and periodic visits by international experts: a 6-day workshop to train and certify health professional leaders in EmOC best practice, audit techniques, and sexual and reproductive rights; a multidisciplinary audit committee established in each facility to undertake a monthly audit according to the WHO guidelines; staff trained in best practice obstetric care; educational outreach sessions every three months and re-certification; international observatories; leadership; supportive supervision; mobilizing financial resources	02 years	Doctors, midwives, and nurses	Referral hospitals (Comprehensive EmOC centres)	A cluster-randomized controlled trial: one pre-intervention year and two intervention years to measure obstetric care quality in the post-intervention year. A criterion-based clinical audit (CBCA) to measure patient history, clinical examination, laboratory examination, birth care, and PNC; reviewing patient charts; *t*-test analysis	Women treated at intervention hospitals have, on average, 5 pp greater CBCA scores than those treated at control hospitals (*β*: 0.052; 95% CI: 0.003–0.102; *p* = 0.04): intervention vs control hospitals: initial interview CBCA scores (82.3% vs 81.1%); first clinical exam CBCA scores (86.4% vs 80.5%; *p* < 0.05); laboratory exams CBCA scores (33.3% vs 31.7%); birth care CBCA scores (63.3% vs 62.8%); postnatal monitoring CBCA scores (56.2% vs 46.1%; *p* < 0.05); significantly more women received good quality care (> 70% criteria attainment): (44.1% vs 29.7%; *p* < 0.001); significantly greater CBCA scores in women treated (68.2 vs 64.5; *p* < 0.05)	Selection: LR Performance: LR Attrition: LR Detection: LR Reporting: LR
Rahman et al. (2011) [51]	Bangladesh	Community involvement in bi-monthly pregnancy surveillance, home-based care through CHWs; health facility-based training on management of normal and complicated deliveries and newborn complications for doctors and midwives, standard guidelines development and implementation for management of maternal and newborn complications; mobilizing financial resources	02 years	Doctors, midwives, and CHWs	Primary care (community-based, healthcare centre), secondary care district hospital, and tertiary care hospitals	Pre- and post-intervention community-based survey at intervention and comparison areas; difference-in-differences analysis	Intervention area: perinatal mortality decreased by odds of 36% as compared to pre-intervention period (AOR: 0.64; 95% CI 0.52–0.78); significant reduction in perinatal mortality in intervention area as compared to the comparison area (p = 0.018); post-intervention area: early pregnancy (GA: 12–14 weeks) ANC home visit: 94.3%, late pregnancy (GA: 32–34 weeks) ANC home visit: 77%; post- vs pre-intervention area: health facility ANC visits (ANC 3 + : 78% vs 38%, ANC 2: 12% vs 43%, ANC 1: 6% vs 15%), health facility birth (72% vs 55%; *p* < 0.001), cesarean section rates (16% vs 8%; *p* < 0.001), < 1 day timing of first newborn bath (4% vs 30%; p < 0.001), colostrum as first newborn food (96% vs 83%; *p* < 0.001); < 30 min timing of first breast feeding (81% vs 61%; *p* < 0.001), preterm births (before 37-week gestation) significantly decreased (12.3% vs 16.8%; *p* < 0.001); intervention vs comparison areas: still birth rate (23/1000 births vs 31/1000 births), early neonatal deaths (17/1000 live births vs 27/ 1000 live births); perinatal mortality rate (3.2% vs 5.6%)	High
Satti et al. (2012) [59]	Rural mountainous Lesotho	Training and performance-based incentives: 3 months training of 100 women, mostly TBAs, to identify pregnant women and accompany them to a health centre for ANC, birth care, and PNC services (clinic-affiliated maternal health workers); deployment of a nurse-midwife to the health centre to provide ANC and birth care and supervise the maternal health workers; public–private sectors partnership	02 years	Traditional birth attendants (TBAs), nurse-midwife	Primary care (community-based and primary healthcare centre)	Before and after secondary data analysis of ANC and delivery registers	The average number of ANC 1 visit increased from 20 to 31 per month; 520 women tested for HIV during the ANC 1 visit, where 94% were with unknown status compared to 18 new PMTCT clients registered in the year preceding the program; VDRL (syphilis) testing for 644 women (86% of ANC 1 visit); haemoglobin testing for 637 women (85% of ANC 1 visit); 218 mothers (122 in year 2) admitted to maternal waiting houses (55% of health facility birth); 178 health facility birth in the 1st year of the program and 216 in the 2nd year, compared to 46 in the year preceding the program; 49 women with complications successfully transferred to the district hospital; no maternal deaths among the women in the program	Moderate
Waiswa et al. (2015) [49]	Rural Uganda	Training CHWs for 5 days on the identification of pregnant women in their community, and undertaking two home visits during pregnancy and three visits after birth at or as close to days 1, 3, and 7 reinforced by directly observed supervision; 6 days in-service training for SABs in 20 public and private health facilities on goal-oriented ANC, managing maternal complications, infection prevention, managing normal labour and partograph use, neonatal resuscitation, care of the sick newborn, and extra care for small babies using kangaroo mother care; community involvement; non-financial incentives (t-shirt, briefcase, certificate); travel refund; mobilizing financial resources	02 years	Community health workers and skilled personnel	Primary care (community-based and primary healthcare facilities)	A cluster-randomized controlled trial: community-based baseline and end-line surveys; t-test analysis (p) for comparison between intervention and control end lines	The interventions provided improved maternal and essential newborn care practices to poorer families—intervention vs control clusters: ≥ 4 ANC visits (47% vs 43.6%; *p* = 0.165); mothers with knowledge of two or more pregnancy-related danger signs (32.7% vs 38%; *p* = 0.126); mothers received ≥ 1 home visit during pregnancy (68.2% vs 7.3%; *p* < 0.001); SPAB (79.6% vs 78.9%; *p* = 0.826); use of TBAs dropped by (5.7% vs 0%); women visited by a CHW in the first week after birth (62.8% vs 5.8%; *p* < 0.001); newborn put to the breast within 1 h of birth (72.6% vs 66%; *p* = 0.0116); newborn given colostrum (93.4% vs 91.2%; *p* = 0.086); baby exclusively breastfed in first month of life (81.8% vs 75.9%; *p* = 0.042); newborn placed skin-to-skin with mother within 1 h of birth (80.7% vs 74.2%; p = 0.071); newborn wrapped immediately after birth (99.6% vs 99.8%; *p* = 0.562); first bath delayed ≥ 24 h after birth (49.6% vs 35.5%; *p* < 0.001); cord cut with clean instrument (88.1% vs 84.4%; *p* = 0.074); nothing applied to umbilical cord after cutting (63.9% vs 53.1%; *p* = 0.002); LBW babies given kangaroo mother care (22.4% vs 9.3%; *p* = 0.089)	Selection: LR Performance: LR Attrition: LR Detection: LR Reporting: LR
Zeng et al. (2018) [60]	Rural Zambia	Results-based and input-based financing; mobilizing financial resources: with the RBF, health facilities were provided with incentives tied to performance on pre-agreed MCH care indicators. Sixty percent of the incentive payment was used for staff bonuses, and 40% was used for operational activities. In IBF, health facilities received funding only for operational activities that were not tied to performance	27 months	Skilled personnel	Primary healthcare facilities	A triple-matched cluster-randomized trial: before and after trial household and facility surveys; difference in Differences (DiD) analysis	RBF districts-DiD: coverages were improved by 19.5% for injectable contraceptives (*p* < 0.05), − 1.5% ANC, 3% IPT in pregnancy (*p* < 0.05), 12.8% SPAB (*p* < 0.01), 8.2% PNC (*p* < 0.05) and 6.1% to 20.4% infant vaccinations as compared to controls; IBF districts-DiD: coverages were improved by − 2.3% for injectable contraceptives, 0% ANC, 0.7% IPT in pregnancy, 17.5% SPAB (*p* < 0.01), 13.2% PNC (*p* < 0.01) and 0.3% to 5.6% infant vaccinations as compared to controls; RBF districts-DiD: coverages were improved by 21.8% for injectable contraceptives (*p* < 0.05), − 1.5% ANC, 2.3% IPT in pregnancy, − 4.9% SPAB, − 5.1% PNC and − 1% to 18.6% infant vaccinations as compared to IBF districts; RBF districts: quality of care index-DiD: improved by 9.7% for injectable contraceptives, 2.9% for ANC, 3.1% for SPAB, 2.3% for PNC and 3.8% for infant vaccinations as compared to the controls; IBF districts: quality of care index-DiD: improved by 4.8% for injectable contraceptives, 2.8% for ANC, 2.4% for SPAB, 3% for PNC and 0.6% for infant vaccinations as compared to the controls; RBF districts: quality of care index-DiD: improved by 4.9% for injectable contraceptives, 0% for ANC, 0.7% for SPAB, − 0.8% for PNC and 3.2% for infant vaccinations as compared to IBF districts; pregnant women and children < 5 years in RBF districts gained 604 and 14,574 QALYs, respectively, while pregnant women and children < 5 years in IBF districts gained 302 and 8,274 QALYs, respectively, as compared to the controls; pregnant women and children < 5 years in RBF districts gained 302 and 6,300 QALYs, respectively, as compared to the IBF districts; incremental cost–effectiveness ratios of US$ 809 and 413 per QALY gained for RBF and IBF districts, respectively, as compared to controls. Incremental cost-effectiveness ratio of US$ 1324 per QALY gained for RBF districts as compared to the IBF districts	Selection: LR Performance: LR Attrition: LR Detection: LR Reporting: LR

The included studies described interventions that had been implemented in a range of settings: primary care (*n* = 13) [39, 40, 44, 47–49, 54–57, 59–61, 63], secondary care hospitals (*n* = 1) [52], primary care, and secondary care hospitals (*n* = 8) [41–43, 45, 46, 50, 53, 58], primary care, and secondary and tertiary care hospitals (*n* = 1) [51], and referral hospitals (*n* = 1) [62].

The interventions involved different cadres of health personnel [16]. Twelve studies included skilled personnel (doctors, nurses, nurse-midwives, midwives, auxiliary nurses, auxiliary midwives, auxiliary nurse midwives, and/or health officers) [39–41, 44, 46, 47, 52, 53, 56, 60–62], three focused on lay personnel (CHWs and/or TBAs) [43, 50, 54]. Five studies included skilled and lay personnel [48, 49, 51, 57, 59], one involved skilled personnel, lay personnel, and lady health supervisors [55]. One study focused on skilled personnel, and healthcare managers [42], one involved skilled personnel, community health supervisors, data officers, and healthcare managers [45], and one study focused on skilled personnel, lay personnel, and healthcare managers [58].

All studies included in the review were intervention studies with 22 collecting quantitative data, one collecting qualitative data, and one collecting mixed data.

Fifteen studies had policy-related interventions [39, 40, 42–44, 46, 48, 49, 52, 54, 57–61], 19 had finance-related interventions [39–46, 48, 49, 51–53, 55, 57, 59–62], 16 had education-related interventions [41, 42, 45, 47–57, 59, 62], 16 had partnership-related interventions [41–45, 48–51, 55–59, 61, 62], 9 had leadership-related interventions [41, 44–46, 48, 50, 53, 58, 62], and 18 had management-related interventions [40, 41, 43, 44, 47–50, 52–59, 61, 62]. Of the 10 studies that had been conducted during the MDGs era, eight featured finance, partnership, and management-related, seven policy-related, and six education-related HRH interventions [40, 44, 46, 48–51, 58, 59, 62], while of 14 studies conducted during the five years SDGs era (2016–2020), 11 papers studied education and finance-related, ten management related, eight policy and partnership related, and three leadership-related HRH interventions[39, 41–43, 45, 47, 52–57, 60, 61]. The HRH interventions that involved education and finance positively affected SRMNH care quality along the continuum. Of the studies conducted during the MDGs era, five investigated HRH interventions implemented to provide SRMNH care quality across ≥ 3 components of the SRMNH care continuum [46, 48–51]. In the studies conducted during the 5 years of the SDGs era, ten featured HRH interventions implemented to provide quality SRMNH care across ≥ 3 components of the SRMNH care continuum [39, 41–43, 45, 53–55, 57, 60]. HRH interventions that had an effect on SRMNH care quality across the continuum are outlined as follows.

### PCC, ANC, IPC, and PNC continuum

Two cluster-randomized trials involving skilled personnel were identified [39, 60]. Engineer et al. [39] described the effect of payment for performance (P4P) on the quality of maternal and child health (MCH) services in Afghanistan. Doctors and mid-level cadres were provided with quarterly bonuses based on the delivery of nine MCH related indicators. There was no direct communication with health workers about the bonuses, nor were there any demand-side interventions (raising or creating demand in communities). The intervention positively affected history taking and physical examinations, and client counseling quality of care indices across the SRMNH care continuum. The intervention, however, had no significant impact on equitable access to the targeted MCH services use between poor and rich families or on the adequacy of time spent with a client along the SRMNH care continuum. It did not also improve client satisfaction and the perceived quality of care along the SRMNH care continuum.

Zeng et al. [60] investigated the effect of results-based financing (RBF), an approach to incentivize healthcare providers and operational activities based on performance, on SRMNH care quality in line with input-based financing (IBF), a traditional approach of increasing funding not tied to performance, in Zambia. Both the RBF and IBF interventions significantly improved the quality of injectable contraceptives, ANC, IPC, and PNC services, respectively, compared to their respective controls (without additional financing). Pregnant women and children in RBF districts gained 604 and 14,574 quality-adjusted life years (QALYs), respectively, while pregnant women and children in IBF districts gained 302 and 8,274 QALYs, respectively, as compared to the controls.

### ANC, IPC, and PNC continuum

Thirteen studies focused on improving SRMNH care quality across ANC, IPC, and PNC continuum. Six studies featured interventions to improve the performance of CHWs. Okuga et al. and Waiswa et al. [48, 49] evaluated the effect of community-based recruitment, training, deployment, supervision, modest financial and non-financial incentives for CHWs, and their integration into the healthcare system on maternal and newborn healthcare in rural Uganda. Health facility strengthening was undertaken at all facilities. Qualitative interviews with key stakeholders found that CHWs were positively received and used their social networks to identify and refer pregnant women and involve men in health education. Okuga et al. (2015) showed reduced delays in healthcare service delivery at health facilities; and improvements in compassionate and respectful care, and cord care. In addition, there were improvements in the early initiation of breastfeeding and feeding newborns on colostrum and delayed bathing of newborns. [48]. Waiswa et al. (2015) found improved maternal and essential newborn care practices among poorer families. Significant, positive impacts of the intervention were identified, including increased health worker home visiting during pregnancy and the first week after birth. There were improvements in early breastfeeding initiation, delayed bathing of newborns (≥ 24 h), and cord care [49].

In the study by Edwards et al. [50], village and community health workers recruited from their respective communities were given skills-based training on maternal and newborn health (MNH) and primary healthcare. They received monthly supportive supervision in rural Bangladesh. Health workers from villages to the general hospital and the community worked in collaboration and team. Confidential, no-blame perinatal and maternal death audit was also implemented. A higher proportion of poor women in intervention areas received ANC, skilled personnel-assisted birth (SPAB), caesarean section, and PNC services than poor women living in the non-intervention, nationally sampled study areas across the SRMNH care continuum. There was also a reduction in the gap in service use between the poorest and richest women in intervention areas than in the national sampled study areas along the SRMNH care continuum. In the study by Agarwal et al. [54], Accredited Social Health Activists (ASHAs) were trained to provide health education and connect women to healthcare facilities and providing home-based ANC and PNC. Exposure to ASHA program compared to non-exposure had no significant effect on completing all services across ANC, IPC, and PNC care continuum.

Mobile technology was the focus of study by Balakrishnan et al. [55] who examined the effect of a mobile phone app (mHealth platform) used by trained community-based frontline health workers (ASHAs, Anganwadi Workers and Auxiliary nurse-midwives) to track services delivered to women and their newborns in India. The intervention villages were found to have an increased uptake of ≥ 90 iron and folic acid tablets during pregnancy, early initiation of breastfeeding, and PNC home visits as compared to the non-intervention control areas. There was equity in the coverage of all quality indicators of SRMNH care across all casts (scheduled vs. others). However, there were no differences between intervention and control areas regarding the uptake of tetanus toxoid injections during pregnancy after a year of intervention. Maru et al. [43] evaluated the effect of a public–private partnership that involved developing an accountable care framework that integrated CHWs through companion and home visits to deliver health facility-based care in rural Nepal. The intervention showed an increase in ≥ 4 ANC visits by 6.4 pp, health facility birth by 11.8 pp, and postnatal contraception by 27.5 pp. Ninety-five percent of pregnant mothers received an ultrasound examination by month 8 or 9 of pregnancy. Infant mortality decreased from 18.3/1000 to 12.5/1000 live births.

Seven studies featured interventions to improve the performance of skilled personnel. Out of these, educational interventions were focused on five studies. Okawa et al. [53] examined the effect of doctors and mid-level cadres training and supervision on adequate contacts and SRMNH care quality in rural Ghana. The intervention had a significant, positive effect on the quality of PNC (*p* = 0.02). The intervention, however, did not significantly improve the quality of ANC or IPC. Having adequate contact with healthcare providers did not guarantee a high quality of care. In the study by McDougal et al. [57], community-based frontline workers were trained, mobilized, and empowered to improve the quality and effectiveness of home visits in India. The intervention had a significant, positive effect for nothing applied to cord after birth, kangaroo mother care, and postnatal contraception use. Ayalew et al. [41] examined the effect of multi-faceted interventions, including the Basic Emergency Obstetric and Newborn Care (BEmONC) training, supportive supervision, audit and site mentoring, and health facility-based quality improvement teamwork in Ethiopia. It had a significant, positive impact on healthcare provider performance during labour and birth (*p* = 0.002) and immediate PNC services (*p* = 0.001) compared to the comparison facilities. Magge et al. [45] studied the effect of clinical mentorship, training, and collaborative district-wide learning and leadership on maternal and newborn care quality in Rwanda. Post- versus pre-intervention outcomes showed pregnant women with premature rupture of membrane (PROM) treated with antibiotics of (38% vs. 24%); pregnant women with preterm labour treated with corticosteroids of (75% vs. 26%); waiting time to C-section in minutes (72 vs. 99); immediate kangaroo mother care (87% vs. 19%); and newborns checked for danger signs within 24 h of birth (98% vs. 47%). Rahman et al. [51] assessed the effect of an integrated packaged interventions, including community participation and onsite training on the management of deliveries and newborn complications on perinatal mortality in Bangladesh. Early pregnancy ANC home visits, caesarean section rates, early initiation of breastfeeding, colostrum as first newborn food, and delayed first newborn bathing were significantly, positively higher in the post-intervention period as compared to two years pre-intervention (*p* < 0.001). In intervention areas, perinatal mortality decreased by odds of 36%; less than 24 h timing of first newborn bath and preterm births significantly decreased.

Two studies examined financial and policy interventions. Binyaruka et al. (2015) and Kambala et al. [42, 46] described the effect of the P4P program (Tanzania) and RBF (rural Malawi) on SRMNH care quality along the continuum. The P4P had a significant, positive impact on one of the eight targeted indicators: anti-malarial treatment during ANC visits (*p* = 0.001). However, there was no evidence of the effect of the P4P program on women's satisfaction with care. There was no significant effect on non-targeted services either (satisfaction with interpersonal care and waiting and consultation times) [46]. Kambala et al. [42] showed that the RBF had no significant, positive impact on women's perceptions of technical care, quality of amenities, or interpersonal relations during ANC, IPC, and PNC. Women reported instances of neglect, disrespect, and verbal abuse by health personnel while receiving care. The health personnel noted an increased workload due to the increased number of women seeking care at intervention facilities.

### ANC and IPC continuum

Six studies focused on improving outcomes across ANC and IPC continuum. Five studies featured interventions to enhance the performance of skilled personnel. Of these, financial and policy interventions were the focus of the two studies. In the study by Basinga et al. [44], the Rwandan government launched a national P4P scheme in health centres based on 14 key MCH-care quality indicators. Quarterly audits of care were made at each health centre based on direct observation and a review of patients' records using a standardized assessment tool on an unannounced, randomly chosen day. The intervention had the greatest effect on indicators that only had the highest payment rates and needed the least effort from the service provider. The intervention had a significant, positive impact on standardized total ANC's quality, the number of high-risk pregnancies referred to district hospital for delivery during pregnancy, and the number of emergency transfers to hospital for obstetric care during delivery. The intervention, however, didn't improve the uptake of tetanus toxoid injections during ANC visits. Bonfrer et al. [40] assessed the effect of the performance-based financing (PBF) policy and quarterly quality assessment by local regulatory authorities on the quality of pregnancy and IPC in Burundi. The PBF policy is the one with financial incentives for healthcare providers based on their performance, excluding operational activities. The program had a significant, positive effect on the coverage of ≥ 1 tetanus toxoid injections during ANC; and SPAB among the non-poor (*p* < 0.028) but the poor in provinces with PBF program as compared to those without it.

Two studies focused on partnership and management interventions. Mwaniki et al. [58] evaluated the effect of a collaborative health worker advisory grouping in improving maternal healthcare quality in rural Kenya. The intervention, at the end line compared to the baseline, had significantly increased ANC contacts per month with standardized care (*p* < 0.001), and pregnant mothers actively referred from the community to health facilities for ANC and IPC services (*p* = 0.012). Duysburgh et al. [61] reported on the effect of a computer-assisted clinical decision support system and performance-based financial and non-financial incentives in Burkina Faso, Ghana, and Tanzania. The intervention showed a significant improvement in the number of lab proteinuria examinations during ANC, history taking on vaginal bleeding during pregnancy, births with correctly completed partograph, blood pressure monitoring during childbirth, and oxytocin for women after childbirth. Larson et al. [47] focused on educational and management interventions in rural Tanzania. Providers from 12 primary care clinics received in-service training, mentoring, supportive supervision and infrastructure support, and community members received peer outreach services. The intervention had a significant, positive, and negative effect on ANC's quality and obstetric care cost, respectively.

The TBA educational and management interventions were featured in a study by Satti et al. [59] in rural Lesotho. In this research, one hundred women, mostly TBAs, were trained and provided with incentives to identify pregnant women in the community and accompany them to a health centre for ANC and delivery. A nurse-midwife was deployed to the health centre to provide care and supervise the TBAs. Pregnant women from isolated communities were accommodated at a maternal waiting room two weeks before delivery. The intervention resulted in that the HIV testing, syphilis testing, and haemoglobin testing during ANC visit increased, as did the number of women with complications who were successfully transferred to the district hospital for obstetric care during delivery. There were no maternal deaths among the women in the program.

### IPC and PNC continuum

Three studies focused on improving outcomes across the IPC and PNC continuum that featured educational and management interventions. Ghosh et al. [56] studied the effect of a multi-faceted, onsite nurse-mentoring and simulation program on nurses' and midwives' skills in the diagnosis and management of intrapartum asphyxia and postpartum haemorrhage (PPH) in India. The mentoring had a significant, positive effect on the diagnosis of asphyxia and PPH and management of asphyxia per additional week of mentoring. Gomez et al. [52] investigated the impact of an onsite, low-dose, high-frequency, and clinical simulation-assisted midwives' BEmONC training in Ghana. The intervention significantly reduced the intrapartum stillbirth and 24-h newborn mortality rates by 52% and 70%, respectively, during the 7–12 months of implementation compared to the baseline. Pirkle et al. [62] evaluated the effect of maternal death reviews in 32 referral hospitals on the quality of obstetrical care in Mali and Senegal. Women treated at intervention hospitals had, on average, higher quality of care scores than those treated at control hospitals.

## Discussion

This study represents the first systematic review to examine HRH interventions and their impact on SRMNH care quality across the continuum in LLMICs. Our findings identified the key elements to successful HRH interventions and the areas for future research regarding HRH interventions.

### Respectful, woman-centred care as a criterion of RBF

The RBF, based on a pre-defined and communicated set of indicators and its policy, significantly improved the quality of targeted SRMNH care services across the continuum given the increasing human resources, equipment, and supply demands are fulfilled [39, 40, 42, 44, 60]. However, these interventions had no significant, positive effect on clients' perception of quality or equity in care [39, 40, 42, 44, 46]. This signals the critical importance of including respectful, woman-centred care. A systematic review suggests that continuous, personalized care provided by the usual midwife and delivered within a family or a specialized setting, generates the highest level of satisfaction [64]. Woman-centred care fosters the woman's psychological and physiological recovery, often surpassing clinical action, and is associated with lower intervention rates [64, 65]. A systematic review from low- and middle-income countries states a positive effect of P4P on history taking, physical examination, blood pressure measurement, and blood and urine testing during ANC visits, and on provider's adherence to explaining medicine intake for children under five years, children are given medicines and children follow-up treatment. However, there was weak evidence for P4P’s positive effect on maternal and neonatal health outcomes and out-of-pocket expenses [66].

### Local budgeting and administration to deliver financial incentives

This review revealed that local budgeting, administration, and policy to deliver financial incentives and improve operational activities, were more effective than mobilizing direct funds from donors for financial incentives, operational activities and incentivizing users, and its policy in improving SRMNH care quality along the continuum and maternal and/or neonatal health outcomes [39, 40, 44, 46, 57, 59, 61]. Reliance on donor funding and donor-driven initiatives may reduce the responsibility for the delivery of care and increase dependence on outside funds. Another study found that despite extensive investment from donors, in-service training and supportive supervision to improve health workers' performance in providing quality antenatal and sick child care in seven countries in sub-Saharan Africa (SSA) did not improve the quality of care [67].

### Training, health worker empowerment, and the integration into the healthcare system

Community-based recruitment, training, deployment, empowerment, supportive supervision, technology assistance, and modest financial and non-financial incentives for CHWs in rural hard-to-reach areas had a significant, positive effect on the quality and equity of SRMNH care services across the continuum. The social networks that CHWs had were a valuable asset enabling the building of rapport and relationships with pregnant women to facilitate referral to nearby health facilities and the provision of home-based life-saving ANC and PNC services [43, 48, 54, 55, 57, 59]. This finding identifies the importance of training for CHWs, and policy initiatives to ensure their empowerment and integration into the healthcare system. A Cochrane trial's review demonstrates that TBA training significantly reduced stillbirth and perinatal and neonatal deaths [22]. There is a need for policy to recognize the important cultural and social roles CHWs fulfill in their local communities that can positively affect maternal and neonatal health outcomes [68].

### BEmONC training, supportive supervision, teamwork, and collaboration for skilled personnel

In-service BEmONC training, supportive supervision, mentoring, audit, quality improvement advisory team, collaboration with CHWs, and community participation for skilled personnel had a significant, positive effect on quality ANC and IPC, and decreased perinatal mortality and preterm births [41, 45, 51, 58]. In a review from Africa, implementing comprehensive interventions that strengthen the health system's different components, both in the community and at the health facilities, directly or indirectly improved the quality of maternal healthcare and morbidity and mortality outcomes [69].

### Skills-based, on-site, regular, and clinical simulation training for nurses and midwives

On-site, short, frequent, regularly run, and clinical simulation-assisted training and mentorship of nurses and midwives had a significant, positive effect on the quality of ANC, IPC, and PNC along the continuum. This, in turn, significantly reduced the intrapartum stillbirth and 24-h newborn mortality rates [45, 52, 56]. On the other hand, in-service training, orientation, mentoring and supportive supervision, and peer outreach services implemented in two studies in support of the continuum-of-care package had only a modest effect on the quality of ANC, IPC, or PNC [47, 53]. Analysis of the Service Provision Assessment data from seven countries in SSA shows that in-service training and supportive supervision had a modest effect on ANC and sick child care quality. Providers were observed performing fewer than half of the recommended clinical actions for pregnant women and sick children on average [67]. Accordingly, skills-based, regular, focused, onsite, and clinical simulation and/or mobile technology-assisted in-service training of skilled personnel are more effective than the knowledge-based, irregular, and ineffective donor-funded training or supervision in LLMICs.

### Maternal and perinatal death audits in all health facilities

Maternal death reviews in 32 referral hospitals in Mali and Senegal, coupled with training and certification of skilled personnel, had a significant, positive effect on ANC and IPC’s quality across the continuum [62]. Confidential, no-blame perinatal and maternal death audits in rural Bangladesh improved the equity for poor women in receiving life-saving maternal healthcare services along the SRMNH care continuum [50]. A systematic review of effective non-drug interventions for improving outcomes and quality of maternal healthcare in SSA also found that facility-based clinical audits and maternal death reviews supported by demand and supply-side financial incentives, health systems strengthening interventions, community mobilization and peer-based programs, and task shifting directly or indirectly improved quality of maternal healthcare and morbidity and mortality outcomes in SSA [69].

### Involving skilled and lay personnel in primary care

In primary care, HRH interventions involving both skilled and lay personnel were more effective than those involving either skilled or lay personnel alone on improving quality or health outcomes [39, 40, 47–49, 55, 61]. A systematic review that focused on IPC found that increasing the use of skilled personnel where TBAs are providers of birth care suggested that deploying midwives closer to communities, financial incentives for providers and users, mobilizing the community, and partnering with TBAs decreased maternal mortality [70].

### Comprehensive HRH interventions targeting different components of the healthcare system

There is no single effective HRH intervention: ≥ 4 comprehensive HRH interventions had a more positive effect on quality SRMNH care continuum and/or health outcomes than those studies with fewer HRH interventions [39, 41, 45, 47–50, 52, 55, 57–59, 62], suggesting a cumulative effect. A systematic review from SSA also indicates that comprehensive HRH interventions implemented at the healthcare system's different components, both in the community and health facilities, improve maternal health [69].

### Inclusion of PCC in the SRMNH care continuum

The PCC, any intervention to optimize a woman's health before pregnancy to improve maternal, newborn, and child health outcomes, effectively improves pregnancy outcomes: including smoking cessation; increased use of folic acid; breastfeeding; greater odds of obtaining ANC; and lower rates of neonatal mortality [71, 72]. This vital component of the SRMNH care continuum lacks our studies reviewed. This review suggests an urgent need to include PCC in the SRMNH care continuum and studies in LLMICs.

The review has several limitations. Despite the inclusion of methodologically moderate- to high-quality intervention studies retrieved from seven databases, some studies may have been missed as our search terms may not have been sufficient to retrieve them. The use of a deductive qualitative content analysis guided by the WHO-HRH action framework may have resulted in the loss of contextual nuances that may have provided insight into the processes that enabled the successful delivery of HRH interventions across the SRMNH care continuum. On the other hand, the number of studies with HRH interventions having a positive effect on SRMNH care quality across the continuum was more than those reporting negative effects, and hence there might be a potential publication bias.

## Conclusions

Policy-makers in LLMICs should include respectful, woman-centred care as a key part of RBF initiatives and ensure healthcare workers' incentives are locally administered and CHWs are integrated into the healthcare system. Skills-based, regularly run, effectiveness focused, onsite, and clinical simulation and/or mobile phone-assisted in-service training of skilled personnel are needed. Besides, facility-based maternal and perinatal death audits in all health facilities, involving skilled and lay personnel, the implementation of ≥ 4 HRH interventions that target the different components of the healthcare system, and the inclusion of PCC in the SRMNH care continuum and studies are recommended.

## Supplementary Information

**Additional file 1.** Tables and additional files.

## Data Availability

Table S1. HRH interventions and areas of intervention. Table S2. Definitions of skilled health personnel and lay health personnel. Table S3. Inclusion criteria. Table S4. Search strategy and keywords. Table S5. Quality of Randomized controlled trials (RCTs). Table S6. Quality of quasi-experimental, prospective (pre/post), post-only and comparison, and post-only studies. Table S7. Quality of qualitative and mixed-method studies.

## Abbreviations

ASHA: Accredited Social Health Activist
ANC: Antenatal Care
BEmONC: Basic Emergency Obstetric and Newborn Care
CHW: Community Health Worker
CBCA: Criterion-based clinical audit
DiD: The difference-in-differences analysis
HRH: Human Resources for Health
IBF: Input-based financing
IPC: Intrapartum care
LLMICs: Low- and lower-middle-income countries
MNH: Maternal and Newborn Health
PNC: Postnatal care
PCC: Preconception care
RCTs: Randomized controlled trials
pp: Percentage points
RBF: Results-based financing
SPAB: Sexual, reproductive, maternal, and newborn health—SRMNHSkilled personnel-assisted birth
SSA: Sub-Saharan Africa
SDGs: Sustainable Development Goals
WHO: World Health Organization
