# Supplementary material for: How can human resources for health interventions contribute to sexual, reproductive, maternal, and newborn healthcare quality across the continuum in low- and lower-middle-income countries? A systematic review
Source: Hum Resour Health. 2021 Apr 21;19:54. doi: 10.1186/s12960-021-00601-3 (PMC8061056; doi:10.1186/s12960-021-00601-3)
Supplement: Supplementary file 1 — Additional file 1. Tables and additional files. [file 12960_2021_601_MOESM1_ESM.docx]

**Table S1. HRH intervention and areas of intervention, WHO.**

| **HRH Intervention** | **Definition** | Areas of intervention |
| --- | --- | --- |
| Policy | Rules, regulations, and legislation for conditions of employment, work standards, and development of the health workforce. | Professional standards, licensing, and accreditation  Authorized scopes of practice for health cadres  Political, social, and financial decisions and choices that impact HRH  Employment law and rules for civil service and other employers |
| Finance | Obtaining, allocating, and disbursing adequate funding for human resources. | Setting levels of salaries and allowances  Budgeting and projections for HRH intervention resource requirements including salaries, allowances, education, incentive packages, etc.  Increasing fiscal space and mobilizing financial resources (e.g., government, Global Fund, PEPFAR, donors)  Data on HRH expenditures (e.g., National Health Accounts, etc.) |
| Education | Production and maintenance of a skilled workforce. | Pre-service education tied to health needs  In-service training (e.g., distance and blended, continuing education)  The capacity of training institutions  Training of community health workers and non-formal care providers. |
| Partnership | Formal and informal linkages aligning key stakeholders (e.g. service providers, sectors, donors, priority disease programs) to maximize the use of resources for HRH. | Mechanisms and processes for multi-stakeholder cooperation (inter-ministerial committees, health worker advisory groups, observatories, donor coordination groups)  Public-private sector agreements  Community involvement in care, treatment, and governance of health services |
| Leadership | Capacity to provide direction, to align people, to mobilize resources, and to reach goals. | Identify, select and support HRH champions and advocates  Capacity for leadership and management at all levels  Capacity to lead multi-sector and sector-wide collaboration  Strengthening professional associations to provide leadership amongst their constituencies |
| HR management systems | Integrated use of data, policy, and practice to plan for necessary staff, recruit, hire, deploy, develop and support health workers. | Personnel systems: workforce planning (including staffing norms), recruitment, hiring, and deployment  Work environment and conditions: employee relations, workplace safety, gender equity, job satisfaction, and career development  HR information system integration of data sources to ensure timely availability of accurate data required for planning, training, appraising, and supporting the workforce  Performance management: performance appraisal, supervision, and productivity  Staff retention: financial and non-financial incentives. |

Table S2. Definitions of skilled health personnel and lay health personnel.

| **Category** | **Definition** | Different names |
| --- | --- | --- |
| Skilled health personnel providing care during childbirth | Competent maternal and newborn health (MNH) professionals educated, trained and regulated to national and international standards. They are competent to 1. Provide and promote evidence-based, human-rights-based, quality, socioculturally sensitive, and dignified care to women and newborns; 2. Facilitate physiological processes during labour and delivery to ensure a clean and positive childbirth experience; and 3. Identify and manage or refer women and/or newborns with complications ([Skilled health personnel, 2018](https://www.who.int/publications/i/item/definition-of-skilled-health-personnel-providing-care-during-childbirth)).  In addition, as part of an integrated team of MNH professionals (including midwives, nurses, obstetricians, paediatricians, and anaesthetists), they perform all signal functions of emergency maternal and newborn care to optimize the health and well-being of women and newborns. (In different countries, these competencies are held by professionals with varying occupational titles). | Assistant medical officer, clinical officer (e.g., in Malawi), medical licentiate practitioner, health officer (e.g., Ethiopia), physician assistant, surgical technician, medical technician non-physician clinician, clinical officer (e.g., in Tanzania, Uganda, Kenya, Zambia), medical assistant, clinical associate, non-physician clinician, auxiliary nurse, nurse assistant, enrolled nurses, auxiliary midwife, registered midwife, midwife, community midwife, family doctor, general practitioners, medical doctor, registered nurse, nurse practitioner, clinical nurse specialist, advance practice nurse, practice nurse, licensed nurse, diploma nurse, BS nurse, nurse clinician, and nurse. |
| Lay health personnel | Any health worker who performs functions related to healthcare delivery; was trained in some way in the context of the intervention; but has received no formal professional or paraprofessional certificate or tertiary education degree (Lewin 2005). | Community health worker, traditional birth attendant, village health worker, treatment supporter, promotors, traditional midwives (Guatemala), dayas (Egypt), dai (Pakistan), and bidan kampong (Malaysia). |

# Table S3. The inclusion and exclusion criteria applied to the screening of studies for the review

| **Included** | **Excluded** |
| --- | --- |
| Explicit management, leadership, partnership, finance, education, and/or policy-related HRH intervention. | Without any of the six health workforce interventions |
| HRH intervention related to SRMNH care services and quality across the continuum | Quality of care for single SRMNH care (either pre-conception, antenatal, intrapartum, or postnatal care service) |
| Skilled and/or lay health personnel | Health care managers and/leaders not directly involved in healthcare services |
| The outcomes were related to maternal and/or neonatal health care. | HRH intervention not related to maternal and/or neonatal health outcomes |
| Primary research | Review studies, discursive or descriptive outlines of projects. |
| The study conducted in low- and lower-middle-income countries | Studies in upper-middle and/or high-income countries. |
| Publication years were ≥2000 | <2000 year of publication |
| The studies were in English | Non-English |

Table S4. Search Strategy and keywords: How can human resources for health interventions contribute to the quality of care in maternal health across the continuum in low-and lower-middle-income countries?

| **PubMed #1 AND #2 AND #3 AND #4 AND #5 = 606** | | | | | | | | | | | | | | | | | | | | |
| --- | --- | --- | --- | --- | --- | --- | --- | --- | --- | --- | --- | --- | --- | --- | --- | --- | --- | --- | --- | --- |
| **Human resources for health intervention (#1)** | | **Quality**  **(#2)** | | | | | **Continuum**  **(#3)** | | | | | | **Maternal health care (#4)** | | | | **Low-and lower-middle-income countries**  **(#5)** | | | |
| "Organization and Administration"[MH] OR "Health Personnel"[MH] OR Health Personnel[TIAB] OR Health workforce[TIAB] OR Health Care Provider*[TIAB] OR Health worker*[TIAB] OR physician[TIAB] OR Doctor[TIAB] OR "Nursing"[MH] OR Nurs*[TIAB] OR Midwi*[TIAB] OR Community Health Worker*[TIAB] OR Community Health Aide*[TIAB] OR Traditional Birth Attendant*[TIAB] OR Intervention[TIAB] OR "Education"[MH] OR Education[TIAB] OR Curricul*[TIAB] OR Educational Measurement*[TIAB] OR Educational Assessment*[TIAB] OR Teaching[TIAB] OR Training[TIAB] OR Capacity Building[TIAB] OR Staff Development[TIAB] OR Human Resources Development[TIAB] OR Partnership[TIAB] OR "Economics"[MH] OR Economic*[TIAB] OR Supervision[TIAB] OR Administration[TIAB] OR Logistics[TIAB] OR Motivation[MH] OR Motivation[TIAB] OR Incentive*[TIAB] OR "Employment"[MH] OR Employment[TIAB] OR Recruitment[TIAB] OR "Leadership"[MH] OR Leadership[TIAB] OR "Intersectoral Collaboration"[MH] OR Intersectoral Collaboration[TIAB] OR Intersectoral Cooperation[TIAB] OR "Community Participation"[MH] OR Community Involvement[TIAB] OR Competency assess*[TIAB] OR "Certification"[MH] OR certificati*[TIAB] OR "Licensure"[MH] OR Licensing[TIAB] OR Skill Mix[TIAB] OR Skills Mix[TIAB] OR Skill-Mix[TIAB] OR Staffing and Scheduling[TIAB] OR Task-Sharing[TIAB] OR Job Sharing[TIAB] OR "Delegation, Professional"[MH] OR Professional Delegation[TIAB] OR Task Shifting[TIAB] OR Night Shift Work[TIAB] OR "Policy"[MH] OR Policy[TIAB] OR Legislation[MH] OR Legislation[TIAB] | | "Health Care Quality, Access, and Evaluation"[MH] OR "Quality of Health Care"[MH] OR Quality[TIAB] OR Optimal Care*[TIAB] OR "Diffusion of Innovation"[MH] OR Innovation Diffusion[TIAB] OR Health Worker Performance[TIAB] OR Care Standard[TIAB] OR Clinical Competenc*[TIAB] OR Protocol Compliance[TIAB] OR Evaluation Stud*[TIAB] OR Outcome Assess*[TIAB] OR Effective health care[TIAB] OR "Cost-Benefit Analysis"[MH] OR Cost Benefit Analysis[TIAB] OR Cost Effectiveness[TIAB] OR Cost-Utility Analysis[TIAB] OR People-centred[TIAB] OR Satisfaction[TIAB] OR "Patient Safety"[MH] OR Patient Safet*[TIAB] OR "Evidence-Based Practice"[MH] OR Evidence Based Practice[TIAB] OR Evidence Based Health Care[TIAB] OR Evidence Based Healthcare[TIAB] OR "Culturally Competent Care"[MH] OR Culturally Competent Care[TIAB] OR Culturally Congruent Care[TIAB] OR Culturally Competent Health Care [TIAB] OR Health Equity[TIAB] | | | | | "Continuity of Patient Care"[MH] OR Continuum of Care[TIAB] OR Care Continuum[TIAB] OR Continuity of Care[TIAB] OR Care Continuity[TIAB] OR "Comprehensive Health Care"[MH] OR Comprehensive Healthcare[TIAB] OR Comprehensive care[TIAB] OR Patient-Centered Care[TIAB] OR Person-Centred Care[TIAB] OR Patient Focused Care[TIAB] OR Primary Health Care[TIAB] OR Primary Healthcare[TIAB] | | | | | | "Reproductive Health Services"[MH] OR Reproductive Health Serv*[TIAB] OR Preconception Care[TIAB] OR Perinatal Care[TIAB] OR "Maternal Health"[MH] OR Maternal Health[TIAB] OR "Maternal Health Services"[MH] OR "Maternal Welfare"[MH] OR Maternal Welfare[TIAB] OR Maternal-Child Health Service*[TIAB] | | | | "Developing Countries"[MH] OR "Developing Countr*"[TIAB] OR "Poverty"[MH] OR "Low-Income Population*"[TIAB] OR Least Developed Countries[TIAB] OR "Less Developed Nation*"[TIAB] OR Low Income Countries[TIAB] OR "Third World Countr*"[TIAB] OR "Third World Nation*"[TIAB] OR Lower Middle Income Countries[TIAB] OR Afghanistan*[TIAB] OR Benin*[TIAB] OR "Burkina Fas*"[TIAB] OR Burundi*[TIAB] OR Central African Republic[TIAB] OR Chad[TIAB] OR Congo[TIAB] OR Eritrea*[TIAB] OR Ethiopia*[TIAB] OR Gambia[TIAB] OR Guinea[TIAB] OR Haiti*[TIAB] OR North Korea[TIAB] OR Liberia*[TIAB] OR Madagascar[TIAB] OR Malawi[TIAB] OR Mali[TIAB] OR Mozambiqu*[TIAB] OR Nepal[TIAB] OR Niger[TIAB] OR Rwanda*[TIAB] OR Sierra Leone*[TIAB] OR Somalia*[TIAB] OR Sudan*[TIAB] OR Syria*[TIAB] OR Tajikistan*[TIAB] OR Tanzania*[TIAB] OR Togo[TIAB] OR Uganda[TIAB] OR Yemen*[TIAB] OR Angola*[TIAB] OR Bangladesh*[TIAB] OR Bhutan*[TIAB] OR Bolivia*[TIAB] OR Cabo Verde[TIAB] OR Cambodia*[TIAB] OR Cameroon*[TIAB] OR Comoros*[TIAB] OR Cote d'Ivoire[TIAB] OR Ivory Coast[TIAB] OR Djibouti*[TIAB] OR Egypt*[TIAB] OR El Salvador*[TIAB] OR Ghana[TIAB] OR Honduras*[TIAB] OR India*[TIAB] OR Indonesia*[TIAB] OR Kenya[TIAB] OR Kiribati*[TIAB] OR Kyrgyz*[TIAB] OR Kirgizstan[TIAB] OR Kirghizia[TIAB] OR Lao PDR[TIAB] OR Laos[TIAB] OR Laos[TIAB] OR Lesotho[TIAB] OR Mauritania*[TIAB] OR Melanesia*[TIAB] OR Micronesia*[TIAB] OR Moldov*[TIAB] OR Mongolia*[TIAB] OR Morocc*[TIAB] OR Myanmar*[TIAB] OR Nicaragua*[TIAB] OR Nigeria[TIAB] OR Pakistan*[TIAB] OR Papua New Guinea[TIAB] OR Philippine*[TIAB] OR Sao Tome and Principe[TIAB] OR Senegal*[TIAB] OR Solomon Islands[TIAB] OR Swaziland*[TIAB] OR Timor-Leste*[TIAB] OR East Timor[TIAB] OR Tunisia*[TIAB] OR Tuvalu*[TIAB] OR Ukrain*[TIAB] OR Uzbekistan*[TIAB] OR Vanuatu*[TIAB] OR Vietnam*[TIAB] OR West Bank[TIAB] OR Gaza[TIAB] OR Zambia*[TIAB] OR Zimbabwe*[TIAB] | | | |
| **Web of Science/Core Collection #1 AND #2 AND #3 AND #4 AND #5 = 503** | | | | | | | | | | | | | | | | | | | | |
| **Human resources for health intervention (#1)** | | | **Quality**  **(#2)** | | | **Continuum (#3)** | | | | | | **Maternal health care (#4)** | | | | | | **Low-and lower-middle-income countries**  **(#5)** | | |
| TS=(Health near/3 Workforce) OR TS=(Health near/3 Personnel) OR TS=(Health* near/3 Force) OR TS=(health* near/3 professional*) OR TS=(Health* near/3 Provider*) OR TS=(Health* near/3 manpower) OR TS=(Health* near/3 Worker*) OR TS=Physician* OR TS=Doctor* OR TS=practitioner* OR TS=Nurs* OR TS=Midwi* OR TS="community health aide*" OR TS="community health officer*" OR TS=(medical near/3 auxiliary) OR TS="traditional birth attendant*" OR TS=Intervention* OR TS=education* OR TS=Residency OR TS=internship OR TS=Training* OR TS=Teaching* OR TS=Curricul* OR TS=(Capacity near/3 Building) OR TS=(Personnel near/3 Management) OR TS=(Human near/3 Development) OR TS=Incentive* OR TS=Staffing OR TS=Scheduling OR TS=Partnership OR TS=Collaboration OR TS=Cooperation OR TS=Financ* OR TS=Budget* OR TS=Supervision OR TS=Motivation* OR TS=Leadership* OR TS=(Community near/3 Participation) OR TS=(Community near/3 Involvement) OR TS=(Competency near/3 Assessment) OR TS=Certificati* OR TS=accreditation* OR TS=Licens* OR TS=(Skill* near/3 Mix*) OR TS=(Task near/3 Sharing) OR TS=(Job near/3 Sharing) OR TS=(Task near/3 Shifting) OR TS=(Shift* near/3 Work*) OR TS=Policy OR TS=Legislation* | | | TS=Quality OR TS=(Optim* near/3 *Care) OR TS=(health* near/3 evaluation*) OR TS=(Standard* near/3 *care) OR TS=Satisfaction OR TS=Safe* OR TS=(*Care near/3 Performance) OR TS=Competen* OR TS=(Guideline near/3 Adherence) OR TS=(Outcome near/3 Assessment) OR TS=(Effective near/3 *care) OR TS=(Cost* near/3 Analysis) OR TS=(Cost near/3 Effective*) OR TS=(People near/3 centred) OR TS=(Evidence near/3 Practice) OR TS=(Evidence near/3 *Care) OR TS=(Cultur* near/3 congruent) OR TS=(*cultural near/3 care) OR TS=Equit* | | | TS=(Continuity near/3 *Care) OR TS=Continuum OR TS=(Comprehensive near/3 *Care) OR TS=(Patient near/3 Care) OR TS=(Person near/3 Care) OR TS=(Primary near/3 *Care) | | | | | | TS=(Reproductive near/3 Health*) OR TS=(Reproductive near/3 *care) OR TS=(Preconception near/3 *Care) OR TS=(Perinatal near/3 *Care) OR TS=(Matern* near/3 Health*) OR TS=(Matern* near/3 *care) OR TS=(Maternal near/3 Welfare) OR TS=(Maternal near/3 Service*) | | | | | | TS=(Low* near/4 Income) OR TS=(Low* near/3 middle) OR TS= "Developing Countr*" OR TS=(Least near/3 Countr*) OR TS="Third World" OR TS=Afghanistan* OR TS=Benin* OR TS= "Burkina Fas*" OR TS=Burundi* OR TS= "Central African Republic" OR TS=Chad* OR TS=Congo* OR TS=Eritrea* OR TS=Ethiopia* OR TS=Gambia* OR TS=Guinea* OR TS=Haiti* OR TS= "North Korea" OR TS=Liberia* OR TS=Madagascar* OR TS=Malawi* OR TS=Mali* OR TS=Mozambiqu* OR TS=Nepal* OR TS=Niger* OR TS=Rwanda* OR TS= "Sierra Leone*" OR TS=Somali* OR TS=Sudan* OR TS=Syria* OR TS=Tajikistan* OR TS=Tanzania* OR TS=Togo* OR TS=Uganda* OR TS=Yemen* OR TS=Angola* OR TS=Bangladesh* OR TS=Bhutan* OR TS=Bolivia* OR TS= "Cabo Verde" OR TS=Cambodia* OR TS=Cameroon* OR TS=Comoros* OR TS= "Cote d'Ivoire" OR TS= "Ivory Coast" OR TS=Djibouti* OR TS=Egypt* OR TS= "El Salvador*" OR TS=Ghana* OR TS=Honduras* OR TS=India* OR TS=Indonesia* OR TS=Kenya* OR TS=Kiribati* OR TS=Kyrgyz* OR TS=Kirghizia* OR TS= "Lao PDR" OR TS=Laos* OR TS=Lesotho* OR TS=Mauritania* OR TS=Melanesia* OR TS=Micronesia* OR TS=Moldov* OR TS=Mongolia* OR TS=Morocc* OR TS=Myanmar* OR TS=Nicaragua* OR TS=Pakistan* OR TS= "Papua New Guinea" OR TS=Philippine* OR TS= "Sao Tome and Principe" OR TS=Senegal* OR TS= "Solomon Islands" OR TS=Swaziland* OR TS= "Timor-Leste*" OR TS= "East Timor" OR TS=Tunisia* OR TS=Tuvalu* OR TS=Ukrain* OR TS=Uzbekistan* OR TS=Vanuatu* OR TS=Vietnam* OR TS= "West Bank" OR TS=Gaza* OR TS=Zambia* OR TS=Zimbabwe* | | |
| **SCOPUS #1 AND #2 AND #3 AND #4 AND #5 = 276** | | | | | | | | | | | | | | | | | | | | |
| **Human resources for health intervention (#1)** | | | **Quality**  **(#2)** | | | | | **Continuum (#3)** | | | | **Maternal health care (#4)** | | | | **Low-and lower-middle-income countries**  **(#5)** | | | | |
| TITLE-ABS("Health workforce") OR TITLE-ABS("Health Personnel") OR TITLE-ABS("Health Care Provider") OR TITLE-ABS("Health worker") OR TITLE-ABS(Physician*) OR TITLE-ABS(Doctor*) OR TITLE-ABS(Nurs*) OR TITLE-ABS(Midwi*) OR TITLE-ABS("Community Health Worker") OR TITLE-ABS("Community Health Aide") OR TITLE-ABS("Village Health Worker") OR TITLE-ABS("Traditional Birth Attendant") OR TITLE-ABS(Intervention*) OR TITLE-ABS(Education*) OR TITLE-ABS(Curricul*) OR TITLE-ABS("Educational Measurement") OR TITLE-ABS("Educational Assessment") OR TITLE-ABS(Teaching*) OR TITLE-ABS(Training*) OR TITLE-ABS("Capacity Building") OR TITLE-ABS("Staff Development") OR TITLE-ABS("Human Resources Development") OR TITLE-ABS("Personnel Management") OR TITLE-ABS(Partnership) OR TITLE-ABS(Economic*) OR TITLE-ABS(Financ*) OR TITLE-ABS(Supervision*) OR TITLE-ABS(Administration) OR TITLE-ABS(Logistics) OR TITLE-ABS(Motivation) OR TITLE-ABS(Incentive*) OR TITLE-ABS(Employment) OR TITLE-ABS(Recruitment*) OR TITLE-ABS(Leadership*) OR TITLE-ABS("Intersectoral Collaboration") OR TITLE-ABS("Intersectoral Cooperation") OR TITLE-ABS("Community Participation") OR TITLE-ABS("Community Involvement") OR TITLE-ABS("Competency assessment") OR TITLE-ABS("Certification") OR TITLE-ABS("Medical Licensure") OR TITLE-ABS(Licens*) OR TITLE-ABS("Supportive Supervision") OR TITLE-ABS("Skill Mix") OR TITLE-ABS("Staffing and Scheduling") OR TITLE-ABS(" Task-Sharing") OR TITLE-ABS("Job Sharing") OR TITLE-ABS("Professional Delegation") OR TITLE-ABS("Task Shifting") OR TITLE-ABS("Shift Work Schedule") OR TITLE-ABS("Night Shift Work") OR TITLE-ABS(Policy*) OR TITLE-ABS(Legislation*) | | | TITLE-ABS(Quality) OR TITLE-ABS("Optimal care") OR TITLE-ABS("Diffusion of Innovation") OR TITLE-ABS("Innovation Diffusion") OR TITLE-ABS("Health Worker Performance") OR TITLE-ABS(Standard) OR TITLE-ABS("Clinical Competence") OR TITLE-ABS("Guideline Adherence") OR TITLE-ABS(Evaluation*) OR TITLE-ABS("Outcome Assessment") OR TITLE-ABS(Effective*) OR TITLE-ABS("Cost Benefit Analysis") OR TITLE-ABS("People Centred") OR TITLE-ABS(Satisfaction) OR TITLE-ABS(Safe) OR TITLE-ABS(Timely) OR TITLE-ABS(Efficient) OR TITLE-ABS(Equitable) OR TITLE-ABS("Evidence-Based Practice") OR TITLE-ABS("Evidence Based Health Care") OR TITLE-ABS("Evidence Based Healthcare") OR TITLE-ABS(Respectful) OR TITLE-ABS("Culturally Congruent Care") OR TITLE-ABS("Culturally Competent") OR TITLE-ABS(Equity) | | | | | TITLE-ABS(Continuity) OR TITLE-ABS(Continuum) OR TITLE-ABS("Transitional Care") OR TITLE-ABS(Comprehensive) OR TITLE-ABS("Patient-Centered Care") OR TITLE-ABS("Person-centred care") OR TITLE-ABS("Primary health care") OR TITLE-ABS("Primary Healthcare") OR TITLE-ABS("Integrated Care") | | | | TITLE-ABS("Reproductive Health Service") OR TITLE-ABS("Preconception Care") OR TITLE-ABS("Perinatal Care") OR TITLE-ABS("Maternal Health") OR TITLE-ABS("Maternity Care") OR TITLE-ABS("Maternal Welfare") OR TITLE-ABS("Maternal-Child Health Service") OR TITLE-ABS("Maternal and Child Health") OR TITLE-ABS("Maternal-Newborn-Child Health") OR TITLE-ABS("Maternal, Newborn, and Child Health") | | | | TITLE-ABS(“Developing Country”) OR TITLE-ABS(“Poor Country”) OR TITLE-ABS(“Low-Income Country”) OR TITLE-ABS(“Least Developed Country”) OR TITLE-ABS(“Less Developed Nation”) OR TITLE-ABS(“Third World Country”) OR TITLE-ABS(“Third World Nation”) OR TITLE-ABS(“lower middle income country”) OR TITLE-ABS(“Low and Lower Middle Income Country”) OR TITLE-ABS(Afghanistan*) OR TITLE-ABS(Benin*) OR TITLE-ABS(“Burkina Fas*”) OR TITLE-ABS(Burundi*) OR TITLE-ABS(“Central African Republic”) OR TITLE-ABS(Chad*) OR TITLE-ABS(Congo*) OR TITLE-ABS(Eritrea*) OR TITLE-ABS(Ethiopia*) OR TITLE-ABS(Gambia*) OR TITLE-ABS(Guinea*) OR TITLE-ABS(Haiti*) OR TITLE-ABS(“North Korea”) OR TITLE-ABS(Liberia*) OR TITLE-ABS(Madagascar*) OR TITLE-ABS(Malawi*) OR TITLE-ABS(Mali*) OR TITLE-ABS(Mozambiqu*) OR TITLE-ABS(Nepal*) OR TITLE-ABS(Niger*) OR TITLE-ABS(Rwanda*) OR TITLE-ABS(“Sierra Leone*”) OR TITLE-ABS(Somalia*) OR TITLE-ABS(Syria*) OR TITLE-ABS(Tajikistan*) OR TITLE-ABS(Tanzania*) OR TITLE-ABS(Togo*) OR TITLE-ABS(Uganda*) OR TITLE-ABS(Yemen*) OR TITLE-ABS(Angola*) OR TITLE-ABS(Bangladesh*) OR TITLE-ABS(Bhutan*) OR TITLE-ABS(Bolivia*) OR TITLE-ABS(“Cabo Verde”) OR TITLE-ABS(Cambodia*) OR TITLE-ABS(Cameroon*) OR TITLE-ABS(Comoros*) OR TITLE-ABS(“Cote d'Ivoire”) OR TITLE-ABS(Djibouti*) OR TITLE-ABS(Egypt*) OR TITLE-ABS(“El Salvador*”) OR TITLE-ABS(Ghana*) OR TITLE-ABS(Honduras*) OR TITLE-ABS(India*) OR TITLE-ABS(Indonesia*) OR TITLE-ABS(Kenya*) OR TITLE-ABS(Kiribati*) OR TITLE-ABS(Kyrgyz*) OR TITLE-ABS(Kirgizstan*) OR TITLE-ABS(Kirghizia*) OR TITLE-ABS(“Lao PDR”) OR TITLE-ABS(Laos*) OR TITLE-ABS(Lesotho*) OR TITLE-ABS(Mauritania*) OR TITLE-ABS(Melanesia*) OR TITLE-ABS(Micronesia*) OR TITLE-ABS(Moldova*) OR TITLE-ABS(Mongolia*) OR TITLE-ABS(Morocco*) OR TITLE-ABS(Myanmar*) OR TITLE-ABS(Nicaragua*) OR TITLE-ABS(Pakistan*) OR TITLE-ABS(“Papua New Guinea”) OR TITLE-ABS(Philippines*) OR TITLE-ABS(“Sao Tome and Principe”) OR TITLE-ABS(Senegal*) OR TITLE-ABS(“Solomon Islands”) OR TITLE-ABS(Sudan*) OR TITLE-ABS(Swaziland*) OR TITLE-ABS(“Timor-Leste*”) OR TITLE-ABS(“East Timor”) OR TITLE-ABS(Tunisia*) OR TITLE-ABS(Tuvalu*) OR TITLE-ABS(Ukraine*) OR TITLE-ABS(Uzbekistan*) OR TITLE-ABS(Vanuatu*) OR TITLE-ABS(Vietnam*) OR TITLE-ABS(“West Bank”) OR TITLE-ABS(Gaza*) OR TITLE-ABS(Zambia*) OR TITLE-ABS(Zimbabwe*) | | | | |
| **CINAHL #1 AND #2 AND #3 AND #4 AND #5 = 167** | | | | | | | | | | | | | | | | | | | | |
| **Human resources for health intervention**  **(#1)** | | | | | **Quality**  **(#2)** | | | | | **Continuum (#3)** | | | | | **Maternal health care (#4)** | | | | | **Low-and lower-middle-income countries**  **(#5)** |
| “Health workforce” OR (MH “Health Personnel+” OR “Health Personnel” OR “Health Care Provider*” OR “Health worker*” OR “Physician*” OR “Doctor” OR MH "Schools, Nursing" OR "Nurs*" OR “Midwi*” OR "Community Health Worker*" OR "Barefoot Doctor*" OR MH "Rural Health Personnel" OR "Village Health Worker*" OR "Traditional Birth Attendant*" OR "Intervention" OR "Education" OR “Curricul*” OR MH "Education, Health Sciences+" OR MH "Cross Training" OR MH "Curriculum+" OR MH "Education, Clinical+" OR MH "Education, Competency-Based" OR MH "Educational Measurement+" OR MH "Teaching+" OR MH "Staff Development+" OR "Inservice Training" OR "Teaching" OR “Training” OR "Capacity Building" OR "Staff Development" OR MH "Management+" OR "Management" OR MH "Consortia+" OR "Partnership" OR "Intersectoral Collaboration" OR “Intersectoral Cooperation” OR MH "Salaries and Fringe Benefits+" OR MH "Reimbursement, Incentive" OR MH "Comparable Worth" OR “Incentive*” OR MH "Economics+" OR "Economics" OR MH "Financing, Organized+" OR “Financ*” OR MH "Clinical Supervision" OR MH "Fieldwork" OR MH "Student Supervision" OR “Supervision” OR MH "Leadership" OR "Leadership" OR MH "Nursing Management+" OR MH "Health Policy+" OR "Policy" OR MH "Practice Acts+" OR MH "Practice Guidelines" OR "Health Legislation" OR “Legislation” OR MH "Public Relations+" OR "Community Participation" OR MH "Community Role" OR MH "Competency Assessment" OR “Competency assess*” OR MH "Certification+" OR "Certification" OR MH "Licensure+" OR MH "Career Mobility+" OR MH "Personnel Selection+" OR MH "Personnel Staffing and Scheduling+" OR “Supportive Supervision” OR “Skill Mix” OR "Task-Sharing" OR “Task Shifting” OR MH "Shiftwork") | | | | | (MH "Quality of Health Care+" OR "Quality of Health Care" OR "Health care quality" OR "Quality of Healthcare" OR "Healthcare Quality" OR "Quality of Care" OR "Optimal Care*" OR MH "Clinical Competence+" OR "Clinical competence" OR MH "Cultural Competence" OR MH "Cultural Safety" OR "Culturally Competent Care" OR MH "National Vocational Qualifications" OR MH "Guideline Adherence" OR "Guideline Adherence" OR MH "Quality Assurance+" OR MH "Quality Assessment+" OR MH "Professional Compliance" OR MH "Outcomes (Health Care)+" OR "Effective health care" OR MH "Cost Benefit Analysis" OR "Cost-Benefit Analysis" OR "Cost Effectiveness" OR MH "Patient Satisfaction+" OR "Patient Satisfaction" OR MH "Patient Safety+" OR "Patient Safety" OR MH "Medical Practice, Evidence-Based" OR "Evidence-Based Practice" OR "Evidence based care" OR "Equity") | | | | | (MH "Continuity of Patient Care+" OR "Continuum of Care" OR "Care Continuum" OR "Continuity of Care" OR "Care Continuity" OR MH "Health Care Delivery, Integrated" OR "Comprehensive Health Care" OR "Comprehensive Healthcare" OR "Comprehensive care" OR MH "Patient Centered Care" OR "Patient-Centered Care" OR MH "Primary Health Care" OR MH "Transitional Care" OR "Primary Healthcare") | | | | | (MH "Reproductive Care (Saba CCC)+" OR "Reproductive Health" OR "Preconception Care" OR "Perinatal Care" OR MH "Perinatal Care (Saba CCC)+" OR MH "Maternal Health Services+" OR MH "Maternal-Child Care+" OR "Maternal Health" OR MH "Obstetric Care+" OR MH "Prenatal Care (Iowa NIC)" OR MH "Delivery, Obstetric+" OR MH "Women's Health" OR MH "Maternal Welfare" OR MH "Maternal-Child Welfare" OR "Maternal Welfare" OR "Maternal-Child Health") | | | | | (MH "Developing Countries" OR "Developing Countr*" OR “Low-Income Population*” OR “Least Developed Countries” OR “Less Developed Nation*” OR MH "Low and Middle Income Countries" OR “Low Income Countries” OR “Third World Countr*” OR “Third World Nation*” OR “Lower Middle Income Countries” OR “Afghanistan*” OR “Benin*” OR “Burkina Fas*” OR “Burundi*” OR “Central African Republic” OR “Chad” OR “Congo” OR “Eritrea*” OR “Ethiopia*” OR “Gambia” OR “Guinea” OR “Haiti*” OR “North Korea” OR “Liberia*” OR “Madagascar” OR “Malawi” OR “Mali” OR “Mozambiqu*” OR “Nepal” OR “Niger” OR “Rwanda*” OR “Sierra Leone*” OR “Somalia*” OR “Sudan*” OR “Syria*” OR “Tajikistan*” OR “Tanzania*” OR “Togo” OR “Uganda” OR “Yemen*” OR “Angola*” OR “Bangladesh*” OR “Bhutan*” OR “Bolivia*” OR “Cabo Verde” OR “Cambodia*” OR “Cameroon*” OR “Comoros*” OR “Cote d'Ivoire” OR “Djibouti*” OR “Egypt*” OR “El Salvador*” OR “Ghana” OR “Honduras*” OR “India*” OR “Indonesia*” OR “Kenya” OR “Kiribati*” OR “Kyrgyz*” OR “Kirgizstan” OR “Kirghizia” OR “Lao PDR” OR “Laos” OR “Laos” OR “Lesotho” OR “Mauritania*” OR “Melanesia*” OR “Micronesia*” OR “Moldova*” OR “Mongolia*” OR “Morocco*” OR “Myanmar*” OR “Nicaragua*” OR “Nigeria” OR “Pakistan*” OR “Papua New Guinea” OR “Philippines*” OR “Sao Tome and Principe” OR “Senegal*” OR “Solomon Islands” OR “Swaziland*” OR “Timor-Leste*” OR “East Timor” OR “Tunisia*” OR “Tuvalu*” OR “Ukraine*” OR “Uzbekistan*” OR “Vanuatu*” OR “Vietnam*” OR “West Bank” OR “Gaza” OR “Zambia*” OR “Zimbabwe*”) |
| **EMBASE/OVID #1 AND #2 AND #3 AND #4 AND #5 = 200** | | | | | | | | | | | | | | | | | | | | |
| **Human resources for health intervention (#1)** | **Quality**  **(#2)** | | | | | **Continuum (#3)** | | | | | **Maternal health care (#4)** | | | | | | **Low-and lower-middle-income countries**  **(#5)** | | | |
| Health workforce.ab,ti. OR Health care personnel/ OR health auxiliary/ OR health care manpower/ OR lay health worker/ OR Health personnel.ab,ti. OR "Physician*".ab,ti. OR "Doctor*".ab,ti. OR "practitioner*".ab,ti. OR "gynaecologist*".ab,ti. OR "obstetrician*".ab,ti. OR exp nurse/ OR "Nurs*".ab,ti. OR exp midwife/ OR "Midwi*".ab,ti. OR "Community Health Worker*".ab,ti. OR "Village Health Worker*".ab,ti. OR traditional birth attendant/ OR "Traditional Birth Attendant*".ab,ti. OR "Education*".ab,ti. OR exp education/ OR Preservice Education.mp. OR Preservice training.mp. OR medical education/ OR curriculum/ OR "Curricul*".ab,ti. OR "Training*".ab,ti. OR in service training/ OR Inservice Training.ab,ti. OR Capacity Building.ab,ti. OR Staff Development.mp. OR personnel management/ OR health care personnel management/ OR "salary and fringe benefit"/ OR skill mix/ OR team building/ OR Skill Mix.ab,ti. OR "Incentive*".ab,ti. OR "Organization and Administration".mp. OR Partnership.mp. OR "Partnership*".ab,ti. OR Intersectoral Collaboration.mp. OR intersectoral collaboration/ OR public-private partnership/ OR finance/ OR Finance.mp. OR "Supervision*".ab,ti. OR Supportive Supervision.mp. OR Employment.mp. OR Leadership.mp. OR leadership/ OR Community Participation.mp. OR community participation/ OR Competency assessment.mp. OR certification/ OR accreditation/ OR Licensing.mp. OR licensing/ OR Task-Sharing.mp. OR Task Shifting.mp. OR health care policy/ OR Health policy.mp. OR Health Legislation.mp. | exp health care quality/ OR health care quality.mp. OR Quality of Health care.mp. OR Quality of Healthcare.mp. OR Quality of Care.mp. OR Optimal Care.mp. OR Quality Improvement.mp. OR total quality management/ OR Diffusion of Innovation.mp. OR mass communication/ OR Standard of Care.mp. OR competence/ OR clinical competence/ OR cultural competence/ OR nursing competence/ OR professional competence/ OR "Competenc*".ab,ti. OR Guideline Adherence.mp. OR protocol compliance/ OR evaluation study.mp. OR program evaluation.mp. OR Outcome assessment.mp. OR Outcome assessment.ab,ti. OR Effective health care.mp. OR Cost-Benefit Analysis.mp. OR "cost benefit analysis"/ OR Cost Effectiveness.mp. OR "cost utility analysis"/ OR economic evaluation/ OR Patient Satisfaction.mp. OR patient satisfaction/ OR safety/ OR patient safety/ OR Safety.ab,ti. OR Evidence-Based Practice.mp. OR Evidence based practice/ OR evidence based medicine/ OR Evidence Based Health Care.mp. OR Culturally Competent Care.mp. OR transcultural care/ OR Health Equity.mp. OR health equity/ | | | | | Continuity of Patient Care.mp. OR exp patient care/ OR Continuum of Care.mp. OR Care Continuum.mp. OR Continuity of Care.mp. OR Care Continuity.mp. OR Comprehensive Health Care.mp. OR health care/ OR Comprehensive care.mp. OR Patient-Centered Care.mp. OR Person-Centred Care.mp. OR Primary Health Care.mp. OR primary medical care/ | | | | | "Reproductive Health Service*".ab,ti. OR Preconception Care.mp. OR prepregnancy care/ OR exp perinatal care/ OR Perinatal Care.mp. OR Maternal Health.mp. OR exp maternal welfare/ OR Maternal Health Service.mp. OR exp maternal health service/ OR "Maternal Health Service*".ab,ti. OR exp maternal care/ OR Maternal-Child Health Service.mp. | | | | | | exp developing country/ OR "Developing Countr*".ab,ti. OR exp lowest income group/ OR Least Developed Countries.mp. OR exp low income country/OR exp lowest income group/ OR "Low-income countr*".ab,ti. OR Third World Countries.mp. OR Lower Middle Income Countries.mp. OR exp middle income country/ OR "Lower-Middle Income Countr*".ab,ti. OR (Low and lower middle income countr*).ab,ti. OR "Afghanistan*".ab,ti. OR "Benin*".ab,ti. OR "Burkina Fas*".ab,ti. OR "Burundi*".ab,ti. OR Central African Republic.ab,ti. OR "Chad*".ab,ti. OR "Congo*".ab,ti. OR "Eritrea*".ab,ti. OR "Ethiopia*".ab,ti. OR "Gambia*".ab,ti. OR "Guinea*".ab,ti. OR "Haiti*".ab,ti. OR North Korea.ab,ti. OR "Liberia*".ab,ti. OR "Madagascar*".ab,ti. OR "Malawi*".ab,ti. OR "Mali*".ab,ti. OR "Mozambiqu*".ab,ti. OR "Nepal*".ab,ti. OR "Niger*".ab,ti. OR "Rwanda*".ab,ti. OR "Sierra Leone*".ab,ti. OR "Somalia*".ab,ti. OR "Sudan*".ab,ti. OR "Syria*".ab,ti. OR "Tajikistan*".ab,ti. OR "Tanzania*".ab,ti. OR "Togo*".ab,ti. OR "Uganda*".ab,ti. OR "Yemen*".ab,ti. OR "Angola*".ab,ti. OR "Bangladesh*".ab,ti. OR "Bhutan*".ab,ti. OR "Bolivia*".ab,ti. OR Cabo Verde.ab,ti. OR "Cambodia*".ab,ti. OR "Cameroon*".ab,ti. OR "Comoros*".ab,ti. OR Cote d'Ivoire.ab,ti. OR Ivory Coast.ab,ti. OR "Djibouti*".ab,ti. OR "Egypt*".ab,ti. OR "El Salvador*".ab,ti. OR "Ghana*".ab,ti. OR "Honduras*".ab,ti. OR "India*".ab,ti. OR "Indonesia*".ab,ti. OR "Kenya*".ab,ti. OR "Kiribati*".ab,ti. OR "Kyrgyz*".ab,ti. OR "Kirghizia*".ab,ti. OR Lao PDR.ab,ti. OR "Laos*".ab,ti. OR "Lesotho*".ab,ti. OR "Mauritania*".ab,ti. OR "Melanesia*".ab,ti. OR "Micronesia*".ab,ti. OR "Moldov*".ab,ti. OR "Mongolia*".ab,ti. OR "Morocc*".ab,ti. OR "Myanmar*".ab,ti. OR "Nicaragua*".ab,ti. OR "Pakistan*".ab,ti. OR Papua New Guinea.ab,ti. OR "Philippine*".ab,ti. OR (Sao Tome and Principe).ab,ti. OR "Senegal*".ab,ti. OR Solomon Islands.ab,ti. OR "Swaziland*".ab,ti. OR "Timor-Leste*".ab,ti. OR East Timor.ab,ti. OR "Tunisia*".ab,ti. OR "Tuvalu*".ab,ti. OR "Ukrain*".ab,ti. OR "Uzbekistan*".ab,ti. OR "Vanuatu*".ab,ti. OR "Vietnam*".ab,ti. OR West Bank.ab,ti. OR "Gaza*".ab,ti. OR "Zambia*".ab,ti. OR "Zimbabwe*".ab,ti. | | | |
| **Cochrane Library/trials #1 AND #2 AND #3 AND #4 AND #5 = 171** | | | | | | | | | | | | | | | | | | | | |
| **Human resources for health intervention**  **(#1)** | | | | **Quality**  **(#2)** | | | | | **Continuum (#3)** | | | | | **Maternal health care (#4)** | | | | | **Low-and lower-middle-income countries**  **(#5)** | |
| ([mh "Health Workforce"] OR [mh ^"Health Personnel"] OR (Health* next Personnel) OR (Health* next *force) OR (health* next professional) OR (Health* next Provider) OR (Health* next worker) OR (Health* next manpower) OR Physician* OR Doctor* OR practitioner* OR Nurs* OR [mh Midwifery] OR Midwi* OR "community health aide" OR "community health officer" OR (medical near/3 auxiliary) OR "traditional birth attendant" OR [mh "Clinical Trial"] OR Intervention* OR [mh Education] OR Education* OR Literacy OR Residency OR Internship OR Training OR Teaching OR Curricul* OR (Capacity near/3 Building) OR (Personnel near/3 Management) OR (Human near/3 Development) OR [mh "Staff Development"] OR [mh ^Motivation] OR Incentiv* OR Staff* OR Scheduling OR [mh "Public-Private Sector Partnerships"] OR [mh "Intersectoral Collaboration"] OR Partnership OR Collaboration OR Cooperation OR [mh "Financial Management"] OR Financ* OR Budget* OR [mh "Organization and Administration"] OR [mh "Inservice Training"] OR Supervision OR Motivation OR Leadership OR [mh "Community Participation"] OR (Community near/3 Participation) OR (Community near/3 Involvement) OR [mh "Competency-Based Education"] OR (Competency near/3 Assessment) OR [mh Certification] OR Certification* OR accreditation* OR [mh Licensure] OR Licens* OR (Skill* next Mix) OR (Task near/3 Sharing) OR (Job near/3 Sharing) OR (Task near/3 Shifting) OR (Shift near/3 Work) OR [mh ^"Health Policy"] OR Policy OR Legislation):ti,ab,kw | | | | ([mh "Quality of Health Care"] OR Quality OR (Optim* next Care) OR [mh "Health Care Evaluation Mechanisms"] OR Evaluation OR Standard* OR [mh "Patient Satisfaction"] OR Satisfaction OR Safe* OR (*Care next Performance) OR [mh "Clinical Competence"] OR [mh "Cultural Competency"] OR Competenc* OR (Guideline near/3 Adherence) OR [mh ^"Outcome Assessment (Health Care)"] OR (Outcome near/3 Assessment) OR [mh "Comparative Effectiveness Research"] OR (Effective next *care) OR [mh "Cost-Benefit Analysis"] OR (Cost near/3 Analysis) OR (Cost next Effective*) OR (People next centred) OR [mh "Evidence-Based Practice"] OR (Evidence near/3 Practice) OR (Evidence near/3 Care) OR (Cultur* next congruent) OR (*cultural next care) OR [mh "Health Equity"] OR Equit*):ti,ab,kw | | | | | ([mh ^"Continuity of Patient Care"] OR (Continuity near/3 Care) OR (Continuum near/3 Care) OR Continuum OR (Comprehensive next *Care) OR (Patient near/3 Care) OR (Person near/3 Care) OR (Primary near/3 Care)):ti,ab,kw | | | | | ([mh "Reproductive Health Services"] OR (Reproductive next Health*) OR (Reproductive near/3 care) OR [mh "Preconception Care"] OR (Preconception next *Care) OR [mh "Perinatal Care"] OR (Perinatal next *Care) OR [mh "Maternal Health"] OR [mh "Maternal Health Services"] OR (Maternal near/5 Health) OR (Matern* next *care) OR [mh "Maternal Welfare"] OR (Matern* next Welfare) OR (Maternal near/5 Service)):ti,ab,kw | | | | | ([mh Poverty] OR (Low* near/5 Income) OR (Low* next middle*) OR [mh "Developing Countries"] OR (Developing next Countries) OR "Least-Developed" OR "Third-World" OR "Under-Developed" OR "Less-Developed" OR Afghanistan* OR Benin* OR "Burkina Faso" OR Burundi* OR "Central African Republic" OR Chad* OR Congo* OR Eritrea* OR Ethiopia* OR Gambia* OR Guinea* OR Haiti* OR "North Korea" OR Liberia* OR Madagascar* OR Malawi* OR Mali* OR Mozambiqu* OR Nepal* OR Niger* OR Rwanda* OR "Sierra Leone*" OR Somali* OR Sudan* OR Syria* OR Tajikistan* OR Tanzania* OR Togo* OR Uganda* OR Yemen* OR Angola* OR Bangladesh* OR Bhutan* OR Bolivia* OR "Cabo Verde" OR Cambodia* OR Cameroon* OR Comoros* OR "Cote d'Ivoire" OR "Ivory Coast" OR Djibouti* OR Egypt* OR "El Salvador*" OR Ghana* OR Honduras* OR India* OR Indonesia* OR Kenya* OR Kiribati* OR Kyrgyz* OR Kirghizia* OR "Lao PDR" OR Laos* OR Lesotho* OR Mauritania* OR Melanesia* OR Micronesia* OR Moldov* OR Mongolia* OR Morocc* OR Myanmar* OR Nicaragua* OR Pakistan* OR "Papua New Guinea" OR Philippine* OR "Sao Tome and Principe" OR Senegal* OR "Solomon Islands" OR Swaziland* OR "Timor-Leste*" OR "East Timor" OR Tunisia* OR Tuvalu* OR Ukrain* OR Uzbekistan* OR Vanuatu* OR Vietnam* OR "West Bank" OR Gaza* OR Zambia* OR Zimbabwe*):ti,ab,kw | |

Date of retrieval for all databases: 21/08/2019; Search date limiter: 01/01/2000

# Methodological quality appraisal

Table S5. Quality of randomized controlled trials (RCTs): Adopted from the revised Cochrane risk of bias (ROB 2.0) tool for randomized trials, 2016.

| **Study** | **Selection bias** | **Performance bias** | **Attrition bias** | **Detection bias** | **Reporting bias** |
| --- | --- | --- | --- | --- | --- |
| Engineer et al.  (RCT) | 1.1. Yes  1.2. Yes  1.3. No  Risk of bias judgement:  Low risk | 2.1. PY  2.2. PY  2.3. No  2.4. NA  2.5. No  2.6. NA  Risk of bias judgement:  Low risk | 3.1. Yes  3.2. NA  3.3. NA  Risk of bias judgement:  Low risk | 4.1. No  4.2. NA  Risk of bias judgement: Low risk | 5.1. No  5.2. No  Risk of bias judgement:  Low risk |
| Gomez et al. (RCT) | 1.1. Yes  1.2. Yes  1.3. Yes  Risk of bias judgement:  Some concerns | 2.1. Yes  2.2. PY  2.3. No  2.4. NA  2.5. No  2.6. NA  Risk of bias judgement:  Low risk | 3.1. Yes  3.2. NA  3.3. NA  Risk of bias judgement:  Low risk | 4.1. No  4.2. NA  Risk of bias judgement: Low risk | 5.1. No  5.2. No  Risk of bias judgement:  Low risk |
| Larson et al. (RCT) | 1.1. Yes  1.2. Yes  1.3. No  Risk of bias judgement:  Low risk | 2.1. No  2.2. NI  2.3. No  2.4. NA  2.5. PY  2.6. No  Risk of bias judgement:  Some concerns | 3.1. Yes  3.2. NA  3.3.NA  Risk of bias judgement: Low risk | 4.1. No  4.2. NA  Risk of bias judgement: Low risk | 5.1. No  5.2. No  Risk of bias judgement:  Low risk |
| Okawa et al. (RCT) | 1.1. Yes  1.2. Yes  1.3. No  Risk of bias judgement:  Low risk | 2.1. PY  2.2. Yes  2.3. No  2.4. NA  2.5. PN  2.6. NA  Risk of bias judgement: Low risk | 3.1. Yes  3.2. NA  3.3. NA  Risk of bias judgement:  Low risk | 4.1. PY  4.2. No  Risk of bias judgement:  Low risk | 5.1. No  5.2. No  Risk of bias judgement:  Low risk |
| Pirkle et al. (RCT) | 1.1. Yes  1.2. Yes  1.3. PN  Risk of bias judgement:  Low Risk | 2.1. PN  2.2. PN  2.3. NA  2.4. NA  2.5. No  2.6. NA  Risk of bias judgement: Low risk | 3.1. Yes  3.2. NA  3.3. NA  Risk of bias judgement:  Low risk | 4.1. PN  4.2. NA  Risk of bias judgement: Low risk | 5.1. No  5.2. No  Risk of bias judgement:  Low risk |
| Waiswa et al. (RCT) | 1.1. Yes  1.2. Yes  1.3. No  Risk of bias judgement: Low risk | 2.1. PN  2.2. Yes  2.3. No  2.4. NA  2.5. No  2.6. NA  Risk of bias judgement: Low risk | 3.1. Yes  3.2. NA  3.3. NA  Risk of bias judgement:  Low risk | 4.1. NI  4.2. No  Risk of bias judgement: Low risk | 5.1. No  5.2. No  Risk of bias judgement:  Low risk |
| Zeng et al.  (RCT) | 1.1. NI  1.2. Yes  1.3. No  Risk of bias judgement: Low risk | 2.1. NI  2.2. PY  2.3. No  2.4. NA  2.5. No  2.6. NA  Risk of bias judgement: Low risk | 3.1. Yes  3.2. NA  3.3. NA  Risk of bias judgement:  Low risk | 4.1. PY  4.2. No  Risk of bias judgement: Low risk | 5.1. No  5.2. No  Risk of bias judgement:  Low risk |

**Signalling questions:**

**1. Selection bias (Randomization process):**

1.1. Was the allocation sequence random?

1.2. Was the allocation sequence concealed until participants were recruited and assigned to interventions?

1.3. Were there baseline imbalances that suggest a problem with the randomization process?

**2. Performance bias (Deviations from intended interventions) - Intention-to-treat (ITT) analysis:**

2.1. Were participants aware of their assigned intervention during the trial?

2.2. Were carers and trial personnel aware of participants' assigned intervention during the trial?

2.3. If Y/PY/NI to 2.1 or 2.2: Were there deviations from the intended intervention beyond what would be expected in usual practice?

2.4. If Y/PY to 2.3: Were these deviations from intended intervention unbalanced between groups and likely to have affected the outcome?

2.5. Were any participants analysed in a group different from the one to which they were assigned?

2.6. Was there potential for a substantial impact (on the estimated effect of intervention) of analysing participants in the wrong group?

**3. Attrition bias (Missing outcome data):**

3.1. Were outcome data available for all, or nearly all, participants randomized?

3.2. If N/PN/NI to 3.1: Are the proportions of missing outcome data and reasons for missing outcome data similar across intervention groups?

3.3. If N/PN/NI to 3.1: Is there evidence that results were robust to the presence of missing outcome data?

**4. Detection bias (measurement of the outcome):**

4.1. Were outcome assessors aware of the intervention received by study participants?

4.2. If Y/PY/NI to 4.1: Was the assessment of the outcome likely to be influenced by knowledge of intervention received?

**5. Bias in reporting (Selection of the reported result):**

Are the reported outcome data likely to have been selected, on the basis of the results, from:

5.1. Multiple outcome measurements (e.g., scales, definitions, time points) within the outcome domain?

5.2. Multiple analyses of the data?

Table S6. Quality of quasi-experimental, prospective (pre/post), post-only and comparison, and post-only studies: Adopted from the JBI Critical Appraisal Checklist for Quasi-Experimental Studies (non-randomized experimental studies).

| **Study** | **1** | **2** | **3** | **4** | **5** | **6** | **7** | **8** | **9** | **Overall quality^*^** |
| --- | --- | --- | --- | --- | --- | --- | --- | --- | --- | --- |
| Agarwal et al. | U | Y | Y | Y | N | NA | Y | Y | Y | 6/9 (Moderate) |
| Ayalew et al. | Y | Y | Y | Y | N | Y | Y | Y | Y | 8/9 (High) |
| Balakrishnan et al. | Y | U | Y | Y | Y | U | Y | Y | Y | 7/9 (High) |
| Basinga et al. | Y | U | Y | Y | Y | U | Y | Y | Y | 7/9 (High) |
| Binyaruka et al. | Y | U | Y | Y | Y | U | Y | Y | Y | 7/9 (High) |
| Bonfrer et al. | Y | Y | Y | Y | N | NA | Y | Y | Y | 7/9 (High) |
| Duysburgh et al. | Y | U | Y | Y | Y | NA | Y | Y | Y | 7/9 (High) |
| Edwards et al. | Y | U | U | Y | N | N | Y | Y | Y | 5/9 (Moderate) |
| Ghosh et al. | Y | Y | Y | N | Y | Y | Y | Y | Y | 8/9 (High) |
| Magge et al. | Y | U | Y | N | Y | U | Y | Y | Y | 6/9 (Moderate) |
| Maru et al. | Y | U | Y | N | Y | U | Y | Y | Y | 6/9 (Moderate) |
| McDougal et al. | Y | Y | Y | Y | Y | NA | Y | Y | Y | 8/9 (High) |
| Mwaniki et al. | U | U | Y | N | Y | Y | Y | Y | N | 5/9 (Moderate) |
| Rahman et al. | Y | N | Y | Y | Y | Y | U | Y | Y | 7/9 (High) |
| Rob et al. | Y | U | U | Y | Y | U | U | N | N | 3/9 (Low) |
| Satti et al. | Y | U | Y | N | Y | U | Y | Y | N | 5/9 (Moderate) |

**Signalling questions:**

1. Is it clear in the study what is the 'cause' and what is the 'effect'

(i.e. there is no confusion about which variable comes first)? Yes (Y)/No (N)/Unclear (U)/ Not Applicable (NA)

2. Were the participants included in any comparisons similar? Y/N/U/NA

3. Were the participants included in any comparisons receiving similar treatment/care, other than the exposure or intervention of interest? Y/N/U/NA

4. Was there a control group? Y/N/U/NA

5. Were there multiple measurements of the outcome both pre and post the intervention/exposure? Y/N/U/NA

6. Was follow up complete and if not, were differences between groups in terms of their follow up adequately described and analyzed? Y/N/U/NA

7. Were the outcomes of participants included in any comparisons measured in the same way? Y/N/U/NA

8. Were outcomes measured in a reliable way? Y/N/U/NA

9. Was appropriate statistical analysis used? Y/N/U/NA

*A study with "yes" responses greater or equal to seven out of nine was considered a high quality, the one scoring four to six "yes" responses was considered a moderate quality and the one scoring less than four "yes" responses was considered a low quality.

# Table S7. Quality of qualitative and mixed method studies: The CASP

| **Signalling questions** | **Kambala et al.** | **Okuga et al.** |
| --- | --- | --- |
| Was the research design appropriate to address the aims of the research? | Yes –& justification provided  Mixed methods-informed by multidimensional definition of Quality | Yes but no overall design/approach or methodology described |
| Was the recruitment strategy appropriate to the aims of the research? | Unclear – participants appear appropriate but no explanation of how they were selected or recruitment processes sampling for diversity focused on intervention sites informed by quant phase | Unclear –description of who took part but not of how they were selected or recruitment processes |
| Was the data collected in a way that addressed the research issue? | Yes but- no rationale for inclusion of interviews and FGDs given | Yes - but- no rationale for inclusion of interviews and FGDs given |
| Has the relationship between researcher and participants been adequately considered? | No | No |
| Have ethical issues been taken into consideration? | Yes | Yes |
| Was the data analysis sufficiently rigorous? | Directed content analysis based on definition of quality Yes | Manifest content analysis sparse detail of analysis process |
| Is there a clear statement of findings? | Yes | Yes |
| How valuable is the research? | Recommendation to local settings but no explicit indication of external validity | Recommendation to local settings and external validity. Yes, valuable |
| Overall comments | Moderate quality | Moderate quality |
